# mTADA is a framework for identifying risk genes from de novo mutations in multiple traits

**DOI:** 10.1038/s41467-020-16487-z

**Published:** 2020-06-10

**Authors:** Tan-Hoang Nguyen, Amanda Dobbyn, Ruth C. Brown, Brien P. Riley, Joseph D. Buxbaum, Dalila Pinto, Shaun M. Purcell, Patrick F. Sullivan, Xin He, Eli A. Stahl

**Affiliations:** 1 Division of Psychiatric Genomics, Department of Genetics and Genomic Sciences, Institute for Genomics and Multiscale Biology, Icahn School of Medicine at Mount Sinai, New York, NY USA; 2 Virginia Institute for Psychiatric and Behavioral Genetics, Department of Psychiatry, Virginia Commonwealth University, Richmond, VA USA; 3 Charles Bronfman Institute for Personalized Medicine, Icahn School of Medicine at Mount Sinai, New York, NY USA; 4 Seaver Autism Center, Department of Psychiatry, Icahn School of Medicine at Mount Sinai, New York, NY USA; 5 The Mindich Child Health & Development Institute, Icahn School of Medicine at Mount Sinai, New York, NY USA; 6 Friedman Brain Institute, Icahn School of Medicine at Mount Sinai, New York, NY USA; 7 Sleep Center, Brigham and Women’s Hospital, Harvard Medical School, Boston, MA USA; 8 Departments of Genetics and Psychiatry, University of North Carolina, Chapel Hill, NC USA; 9 Department of Human Genetics, University of Chicago, Chicago, IL USA; 10 Grossman Institute for Neuroscience, Quantitative Biology and Human Behavior, University of Chicago, Chicago, IL USA; 11 Stanley Center for Psychiatric Research, Broad Institute of MIT and Harvard, Cambridge, MA USA

**Keywords:** Genome informatics, Genetic association study, Neurodevelopmental disorders, Cardiovascular genetics

## Abstract

Joint analysis of multiple traits can result in the identification of associations not found through the analysis of each trait in isolation. Studies of neuropsychiatric disorders and congenital heart disease (CHD) which use de novo mutations (DNMs) from parent-offspring trios have reported multiple putatively causal genes. However, a joint analysis method designed to integrate DNMs from multiple studies has yet to be implemented. We here introduce multiple-trait TADA (mTADA) which jointly analyzes two traits using DNMs from non-overlapping family samples. We first demonstrate that mTADA is able to leverage genetic overlaps to increase the statistical power of risk-gene identification. We then apply mTADA to large datasets of >13,000 trios for five neuropsychiatric disorders and CHD. We report additional risk genes for schizophrenia, epileptic encephalopathies and CHD. We outline some shared and specific biological information of intellectual disability and CHD by conducting systems biology analyses of genes prioritized by mTADA.

## Abstract (web summary)

Joint analysis of multiple traits can increase power and provide insights into shared genetic architecture. Here, Nguyen et al. develop multi-trait TADA (mTADA), an extension of TADA (transmission and de novo association test) that jointly analyses de novo mutations of traits for improved risk-gene identification power.

## Introduction

The analysis of multiple traits can help characterize the genetic architectures of complex disorders^1^. One approach is to meta-analyze results derived from separate single-trait studies^2^. However, joint analysis of multiple traits can better accommodate heterogeneity of genetic effects of the same variants or genes across traits^3,4^. Numerous studies have jointly analyzed two or more traits and successfully identified shared common-variant associations^5–8^. In addition, additional risk loci have been identified using these approaches^7,9^. However, none of these studies has examined rare variation from case-control data, or de novo variants for which mutation rates should be taken into account. For these rare variants, gene based tests have identified several genes associated with different disorders^10–13^. Some recent studies have shown that there are multiple risk genes that are shared between neurodevelopmental disorders^10,14,15^, and also with congenital heart disease (CHD)^16,17^. These results are based on the intersection among the top prioritized genes from each disorder; therefore, reported numbers of genes shared by multiple disorders remain low^10,17^. Development of multi-trait rare-variant methods for neuropsychiatric disorders (NPDs) and related disorders will facilitate the understanding of this important aspect of genetic architecture for these phenotypes.

Currently, there is still a limitation in the risk gene identification for a single trait of NPDs and relevant disorders from parent-offspring trio studies. One reason is that risk gene discovery is underpowered when sample sizes are limited, as well as when relative risks are not large^10,11^. Multiple risk genes have been reported for undiagnosed developmental disorders (DD), intellectual disability (ID) and autism spectrum disorder (ASD)^12,18,19^ thanks to large sample sizes and/or relative risks^10^. However, there are a few risk genes identified for schizophrenia (SCZ), epileptic encephalopathies (EE) and other disorders because of small gene-level relative risks or small sample sizes^10,20,21^. Increasing sample sizes will increase power to identify additional risk genes, but this is an expensive solution and may not be feasible for some studies. If there are genetic overlaps, methods that can leverage the information from one trait to increase power for risk-gene identification of another trait could help in obtaining additional genes for these disorders.

Here, we have developed a new statistical model, mTADA (multi-trait transmission and de novo association test), that jointly analyzes de novo mutations (DNMs) of two traits in order to estimate the gene-level genetic overlap of the two traits, and to identify additional risk genes for each analyzed trait as well as shared and specific risk genes. First, we utilize simulation data and demonstrate that, compared with a single-trait method, mTADA substantially improves the power of risk-gene identification when genetic overlaps increase, especially for traits with smaller sample sizes or smaller relative risks. For example, mTADA is able to statistically increase evidence for multiple genes in a tested trait which shows 1) marginally statistical evidence in that trait, and 2) strong evidence in the other trait if the two traits have a high genetic overlap. To illustrate the advantage of the new pipeline over its previous single-trait version, we apply the method to large data sets of different NPDs and CHD (>13,000 parent-offspring trios) and identify shared genes between each pair of these disorders. mTADA identifies additional risk genes for each disorder by borrowing the information of other traits. We validate these results in an independent cohort of 1,241 trios with CHD, 197 trios with EE, and 4,877 SCZ cases and 6,203 controls. In addition, we demonstrate that mTADA’s results could be used to better understand the shared and specific biological information for two tested disorders by using multiple systems biology approaches to test the top prioritized risk genes of the CHD-ID pair. CHD-specific genes are specific to certain biological pathways.

## Results

### The mTADA framework

**Fig. 1:**
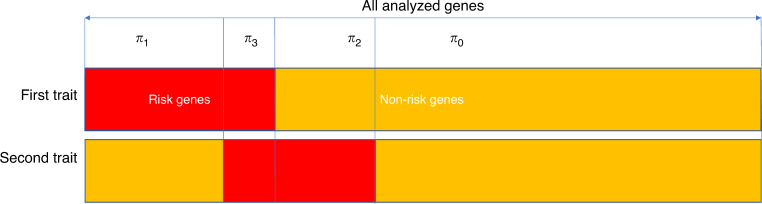
The multiple trait transmission and de novo association test (mTADA). For each trait, mTADA divides the all tested genes into two sets: risk and non-risk genes. Therefore, there are four sets when two traits are combined: risk genes for neither of traits (H_0_), for the first trait only (H_1_), for the second trait only (H_2_), and for both traits (H_3_). Statistical details of four models for these four hypotheses are described in Table 1. *π*_j_ (*j* = 0..3) are prior probabilities for the four models. From mTADA’s analysis results, each gene has four posterior probabilities (PPs) of the four models (e.g., PP0, PP1, PP2 and PP3 for Model 0, Model 1, Model 2 and Model 3 respectively).

The mTADA method is gene-based and requires input data of the number of DNMs and mutation rate per gene. If the DNMs are stratified on the basis of predicted effect (e.g., ‘missense’, ‘nonsense’, etc.), then each gene-annotation category should have its own mutation rate that reflects the predicted effects of the mutations within. In summary, for each gene, we consider four models *M*_*j *_(*j* = 0..3) reflecting four alternative hypotheses: the gene is associated with neither trait (H_0_), the first trait only (H_1_), the second trait only (H_2_), or both traits (H_3_). We assume prior probabilities *π*_*j*_ (*j* = 0..3) for the four models and these π_j_ are estimated from data and single-trait studies. DNMs are modeled using Poisson distributions with mean relative risks, mutation rates and sample sizes as main parameters^10^ (Methods). For each gene, four posterior probabilities (PP), which are abbreviated as PP0, PP1, PP2 and PP3 respectively, are used to infer the status of the gene for the four models. To summarize the evidence for association with a given trait, we use the sum of PPs of models including the risk gene hypotheses for that trait, i.e., PP1 + PP3 for trait one and PP2 + PP3 for trait two (Fig. 1, Table 1, Methods).

**Table 1:** Statistical models of mTADA.

Hypothesis	Proportion	First trait	Second trait
*H*_0_	*π*_0_	$x_{i1}\sim Poisson\left( {2N_1\mu _i} \right)$	$x_{i2}\sim Poisson(2N_2\mu _i)$
*H*_1_	*π*_1_	$x_{i1}\sim Poisson(2N_1\mu _i\gamma _{i1})$ $\gamma _{i1}\sim Gamma(\bar \gamma _1\beta _1,\beta _1)$	$x_{i2}\sim Poisson(2N_2\mu _i)$
*H*_2_	*π*_2_	$x_{i1}\sim Poisson(2N_1\mu _i)$	$x_{i2}\sim Poisson(2N_2\mu _i\gamma _{i2})$ $\gamma _{i2}\sim Gamma(\bar \gamma _2\beta _2,\beta _2)$
*H*_3_	*π*_3_	$x_{i1}\sim Poisson(2N_1\mu _i\gamma _{i1})$ $\gamma _{i1}\sim Gamma(\bar \gamma _1\beta _1,\beta _1)$	$x_{i2}\sim Poisson(2N_2\mu _i\gamma _{i2})$ $\gamma _{i2}\sim Gamma\left( {\bar \gamma _2\beta _2,\beta _2} \right)$

Statistical models for four hypotheses in mTADA for one category of variants in each trait at the i^th^ gene. mTADA assumes that the gene can be in one of four models M_0_..M_3_. *π*_j_ (*j* = 0..3) is the prior probability of the j^th^ model. x_k_ and N_k_ (*k* = 1, 2) are the data and the sample size of the k^th^ trait. μ_i_ is the mutation rate of the gene. For each trait, the relative risks of shared and specific genes (γ_k_) are from a Gamma distribution with two parameters: $\bar \gamma _k$ (mean relative risk) and β_k_ (to control the variance of relative risks).

### Results of mTADA on simulated data

To validate the new method, we conducted simulation studies by using genetic parameters from real-data analyses of previous studies (Methods).

**Fig. 2:**
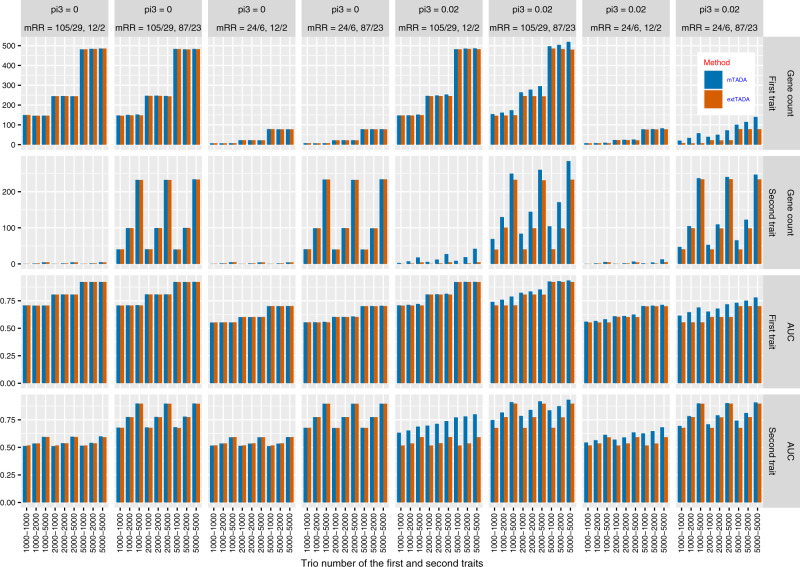
Comparison results of simulated data for the current multi-trait approach (mTADA) and a previous single-trait approach (extTADA) in single-trait analyses. For each bar, its height shows the average value of 100 simulations. mTADA performs better than extTADA when the proportions of overlapping risk genes (pi3) are larger than zero. The top two lines describe gene counts (posterior probability >0.8, while the two bottom lines show area under the Receiver Operating Characteristic (ROC) curves (AUCs). mRR describes mean relative risks and the trio number along the bottom describes the sample sizes. These results are for two variant categories. For example, “mRR = 105/29, 12/2” describes the mRRs of the first trait as 105 and 29, and the mRRs of the second trait as 12 and 2.

*Power for single-trait risk gene discovery.* We compared gene numbers identified by mTADA and our previous single-trait method, extTADA, using the same threshold PP > 0.8. For *π*_3_ = 0 (no overlapping information), mTADA and extTADA reported nearly the same positive gene numbers (Fig. 2). However, mTADA identified more genes than extTADA when *π*_3_ increased. In addition, mTADA’s gene counts were also higher than those of extTADA when higher mean relative risks were used.

*Comparison of risk-gene classification for single traits.* We designed a simulation experiment to assess the performance in the classification of risk and non-risk genes. We applied extTADA to single-trait data from our simulated data. We then calculated areas under the Receiver Operating Characteristic curves (AUCs) for mTADA and extTADA using classification results from single-trait data. AUCs of both were equal when *π*_3_ = 0 (Fig. 2). However, AUCs of mTADA were higher than those of extTADA when *π*_3_’s values were larger. In addition, mTADA also performed better than extTADA with larger mean relative risks.

**Fig. 3:**
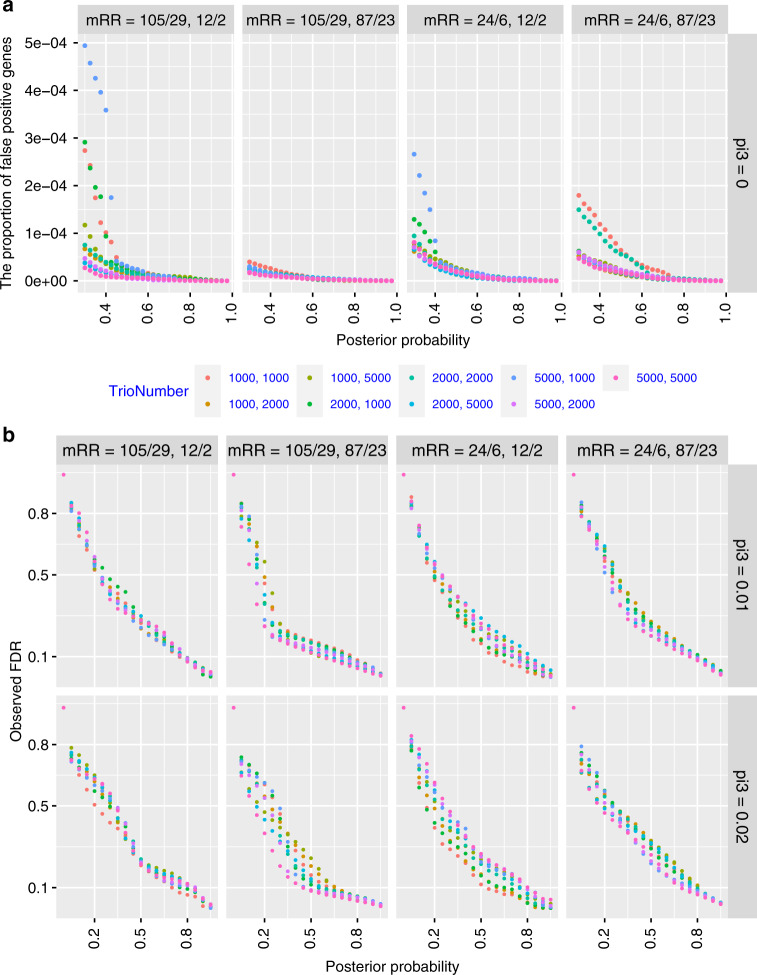
Validation of shared risk gene identification using mTADA on simulated data. **a** The proportion of false positive genes (per 19,358 analyzed genes): *X*-axes are posterior probabilities of Model III while *Y*-axes are the proportions of false positive shared risk genes. **b** The correlation between posterior probabilities (*x*-axis) and observed false discovery rates (FDRs, *y*-axis). These are for the combination of different sample sizes (ntrio) and mean relative risks (mRR).

*The proportion of false positive shared risk genes for two traits with non-genetic overlaps.* We estimated this information for identifying shared risk genes (i.e. associated with both traits). We simulated data with *π*_3_ = 0 and calculated the proportion of shared risk genes (per 19,358 tested genes) using different PP thresholds of Model III (PP3). Overall, these proportions were very small for different PP thresholds (<4.5 × 10^−4^, Fig. 3a).

*Correlations between posterior probabilities and observed false discovery rates (FDRs).* Since mTADA makes inference on risk genes using PPs, instead of commonly used FDR, we compared these two metrics. We calculated the correlation between PPs and observed false discovery rates (oFDRs) for all situations. For PP3, we found that PP = 0.8 and 0.5 approximately correspond to oFDR = 0.1 and 0.25, respectively. Small mean relative risks could lead to higher FDRs, but this inflation was modest (Fig. 3b). These results were similar for other situations: when genes were associated with only the first trait, only the second trait, single traits (e.g., Trait 1 or Trait 2 genes) (Supplementary Figs. 1, 2).

The correlation between simulated and estimated values of *π*_3_ was also assessed. For large mean relative risks, high correlations were observed for all sample sizes. For smaller mean relative risks (≤24), *π*_3_’s values were over- or underestimated (Supplementary Fig. 3). However, these small differences did not affect the results of risk-gene identification (Fig. 2, Supplementary Figs. 1, 2).

*The effects of misdiagnosis and ascertainment bias on the results.* When sample phenotypes are misdiagnosed (a patient of one trait is mis-assigned to another), the estimated parameters of mTADA may be biased and this may affect the results. In another scenario, samples from one trait may contain a larger number of patients of the second trait than expected based on the comorbidity in the population. This ascertainment bias may also have an effect on mTADA’s estimates. We tested the impact of these scenarios. Overall, *π*_3_ and downstream results were not strongly affected when there was ascertainment bias. Similar results were also observed for misdiagnosis rates of 5–10% if the mean relative risks of the tested traits were not highly imbalanced. If the mean relative risks of one trait were substantially higher than those of the other trait, overestimation of *π*_3_ might arise for misdiagnosis rates of ≥5%. Detailed results are in the Supplementary Note 1.

### Application of mTADA to neuropsychiatric disorders and CHD data

**Fig. 4:**
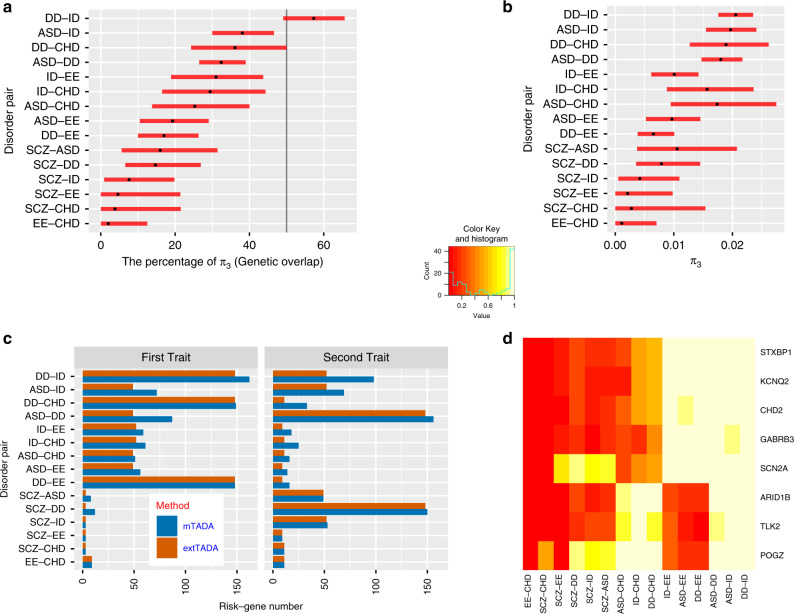
Analysis results of mTADA for pairs of disorders. **a** The estimated gene-level genetic overlaps (gOs) of pairs of disorders from Markov Chain Monte Carlo sampling results. Each par shows the credible interval and the black dot is the estimated value. The vertical black line describes g0 = 50%. **b** The estimated proportion of overlapping risk genes (π_3_) in the mTADA model. **c** Comparison of mTADA and extTADA in the prioritization of top genes by using a threshold of posterior probability (PP) > 0.8. In mTADA, the column ‘First trait’ and ‘Second trait’ are inferred by summing the PPs of model 1 and 3 (PP1 + PP3), and model 2 and model 3 (PP2 + PP3) in Fig. 1 respectively. **d** These genes appear in at least 4 pairs of disorders (PP > 0.8). Cells show the PP values. *Y*-axis shows gene names and *x*-axis describes pairs of disorders.

mTADA was applied to DNM datasets of 15 pairs of six disorders: five neuropsychiatric disorders including DD, ID, ASD, SCZ, EE; and CHD. These DNMs were classified into different categories using annotation tools. Based on previous results^10,22^, we used loss of function (LoF), missense damaging (MiD) DNMs for all disorders and also added synonymous DNMs within DNase I hypersensitive sites for SCZ (Methods). We defined the gene-level genetic overlap (*gO*) of two disorders as: $gO = 100\% \times \pi _3{\mathrm{/}}(\pi _1 + \pi _2 + \pi _3)$. DD based results showed strong convergence with smaller credible intervals (CIs) because of its large sample size as well as high relative risks of DNMs (Fig. 4a/b, Supplementary Table 1). As expected, high *gOs* were observed for pairs of DD, ID, and ASD ($gO \,> \, 32\% ,\pi _3 \,> \, 0.018$). CHD and EE had the lowest *gO* (*gO* = 2%, *π*_3_ = 0.001) followed by SCZ-EE (*gO* = 4.6%, *π*_3_ = 0.0023). Supplementary Fig. 4 shows sampling results of the proportions of overlapping risk genes for pairs of these traits, and Supplementary Fig. 5 shows the percentage of genetic overlaps for traits. The *gO* of ASD and SCZ which was approximately 16% (CI = 5.6–31.4%) was similar to previous studies (Supplementary Table 2).

We also compared mTADA and extTADA in the identification of risk genes for single traits using a threshold of PP > 0.8. For DD and ID, mTADA always performed better than extTADA (Fig. 4c). Similar results were observed for ASD; except for the pair ASD-SCZ in which mTADA was slightly better than extTADA for SCZ but extTADA was better than mTADA for ASD. For CHD, EE and SCZ, mTADA was better than extTADA when CHD was combined with DD.

### Insights into top genes prioritized by mTADA

To better understand the top genes prioritized by mTADA, we extracted genes with PP > 0.8 for further analyses.

*Overlapping genes between two traits.* The highest number of overlapping genes was observed for DD and ID (89 genes) followed by ASD-DD (65 genes) and ASD-ID (47 genes). Four pairs of traits (CHD-EE, SCZ-EE, CHD-SCZ, SCZ-ID) had no overlapping genes. 152 genes were supported by the two-trait model in at least one pair (PP3 > 0.8, Supplementary Data 1). Eight genes (*ARID1B*, *GABRB3*, *KCNQ2*, *STXBP1*, *CHD2*, *TLK2*, *POGZ*, *SCN2A*) were observed for at least six pairs of disorders (Fig. 4d). *POGZ* and *SCN2A* were present in eight pairs of disorders. *POGZ* was significant for pairs relating to ASD, DD, ID, CHD and SZ while *SCN2A* was significant for pairs relating to ASD, DD, EE, ID and SCZ. We checked DNMs of these two genes. As expected, *POGZ* had no DNMs for EE, and *SCN2A* had no DNMs for CHD. Interestingly, in the latest CHD study^23^, *POGZ* was one of the top CHD genes while no DNMs were observed for *SCN2A*. In addition, in a recent study of 6,753 parent–offspring trios with neurodevelopmental disorders and epilepsy^24^, 16 DNMs were in *POGZ*, but only one DNM was from a patient who has both ID and epilepsy.

*Significant genes of single traits.* To demonstrate the application of mTADA in the identification of additional risk genes, we tested three disorders (CHD, EE, and SCZ) whose DN-based genes have not been reported as often as the three other disorders. We used DD-based results because the number of risk genes for the three disorders highly increased when their datasets were jointly analyzed with the DD dataset in Fig. 4c.

**Fig. 5:**
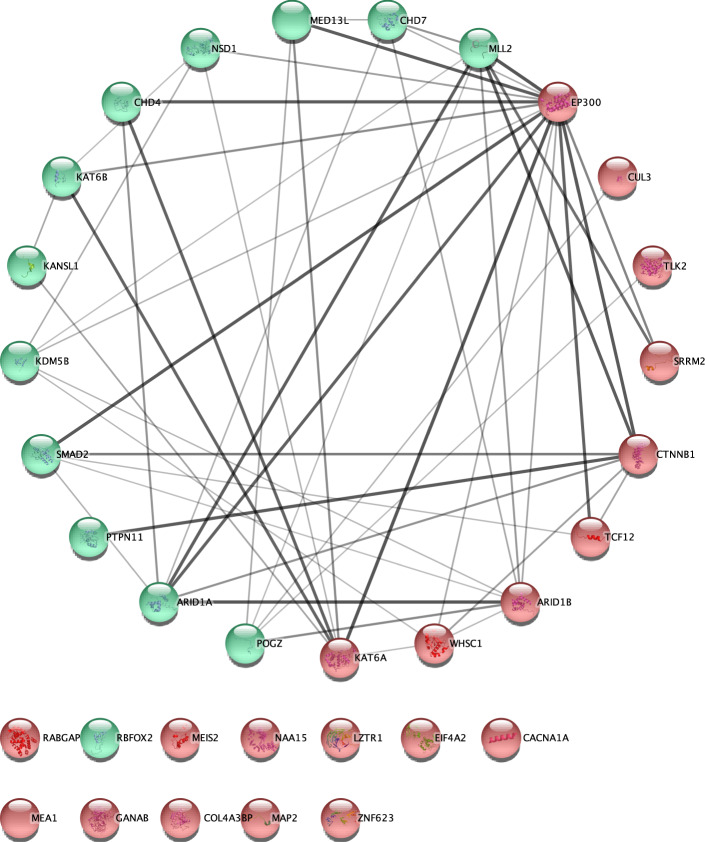
Result of protein-protein interaction analysis for genes associated with congenital heart disease (CHD). These genes were prioritized by using undiagnosed developmental disorders (DD) information. This is the top 33 genes, posterior probabilities > 0.8, identified by mTADA using the data set of Homsy et al.^16^. Novel genes have red background and known genes have green background. Additional information for these genes is in Table 2.

*CHD.* 33 genes were prioritized. 20/31 were not in the list of known CHD genes and in the meta-analysis results of a recent CHD study of Jin et al.^23^ (Table 2). We validated these results by using different approaches. First, we tested the protein-protein interactions (PPIs) of these 33 genes by using the STRING database^25^. The number of edges was higher than expected between 33 protein nodes (PPI *p* = 6e-12, Fig. 5). Multiple protein products of novel and known genes interacted with each other. The number of interactions decreased when tested with only PPIs from experiments but was still significant (PPI *p* = 0.0174). Second, we tested these CHD genes from an independent data set which includes 1,241 trios and 226 cases^23^. From the 1,241 trios, three genes (*CTNNB1*, *CUL3*, *LZTR1*) of the 20 novel genes had LoF or MiD DNMs (Poisson-test *p* < 2.0e-4, Table 2). Each of these three genes had only one DNM in the primary analysis. In addition, these genes were not called significant genes by extTADA. Finally, we compared these 33 genes with the top 25 genes meta-analyzed by Jin et al.^23^. 8/33 were in the 25-gene list (permutation *p* < 9.99e-05; Table 2).

**Table 2:** Information of genes prioritized for congenital heart disease (CHD).

*Gene*	dn_MiD_DD	dn_LoF_DD	dn_MiD_CHD	dn_LoF_CHD	PP	dn_MiD_CHD2018	dn_LoF_CHD2018	pPoisson	Known_Gene	Top 25 genes from Jin et al., 2018
*KDM5B*	0	3	0	3	1	0	0	1.00E+00	N	2.90E-04
*MLL2*	0	2	0	4	1	0	7	5.50E-15	Y	8.50E-19
*NAA15*	0	2	0	2	1	0	0	1.00E+00	N	–
*CHD7*	2	2	0	2	1	3	9	3.98E-24	Y	7.50E-19
*RBFOX2*	0	0	0	3	1	0	0	1.00E+00	N	1.10E-06
*PTPN11*	2	0	3	0	1	2	0	1.17E-04	Y	1.80E-15
*POGZ*	0	6	1	1	1	0	1	1.32E-02	N	2.90E-04
*CTNNB1*	0	11	0	1	0.97	0	1	1.90E-02	N	–
*TCF12*	1	2	0	1	0.97	0	0	1.00E+00	N	–
*KANSL1*	0	8	0	1	0.97	0	0	1.00E+00	Y	–
*MEIS2*	0	2	0	1	0.97	0	0	1.00E+00	N	–
*EIF4A2*	1	1	0	1	0.97	0	0	1.00E+00	N	–
*WHSC1*	0	3	0	1	0.97	0	0	1.00E+00	N	–
*KAT6B*	0	8	0	1	0.97	0	0	1.00E+00	Y	–
*MAP2*	0	2	0	1	0.97	0	0	1.00E+00	N	–
*CUL3*	0	2	0	1	0.97	0	1	2.21E-02	N	–
*ARID1B*	0	30	0	1	0.97	0	0	1.00E+00	N	–
*KAT6A*	0	8	0	1	0.97	0	0	1.00E+00	N	–
*NSD1*	1	7	0	1	0.96	0	2	7.82E-04	Y	1.30E-04
*EP300*	3	12	0	1	0.96	0	0	1.00E+00	N	–
*CACNA1A*	5	0	0	1	0.95	0	0	1.00E+00	N	–
*MEA1*	0	1	0	1	0.95	0	0	1.00E+00	N	–
*ZNF623*	0	1	0	1	0.94	0	0	1.00E+00	N	–
*GANAB*	2	0	1	1	0.94	0	0	1.00E+00	N	–
*COL4A3BP*	4	0	1	0	0.92	0	0	1.00E+00	N	–
*LZTR1*	2	1	1	0	0.91	1	0	2.65E-02	N	–
*RABGAP1L*	0	1	1	1	0.9	0	0	1.00E+00	N	–
*MED13L*	5	13	1	0	0.89	0	0	1.00E+00	Y	–
*TLK2*	2	0	0	1	0.87	0	0	1.00E+00	N	–
*ARID1A*	1	2	1	0	0.87	0	0	1.00E+00	Y	–
*SRRM2*	0	2	0	1	0.87	0	0	1.00E+00	N	–
*CHD4*	5	1	1	0	0.86	1	0	7.30E-02	Y	–
*SMAD2*	0	0	1	1	0.8	0	0	1.00E+00	N	1.60E-04

These 33 genes are prioritized by mTADA using the information of undiagnosed developmental disorders (DD). Columns ‘dn_LoF/MiD’ describe the number of loss-of-function/missense damaging de novo mutations. mTADA was applied to the DD and the CHD data in the 2^nd^, 3^rd^, 4^th^ and 5^th^ columns. The column ‘PP’ describes the posterior probabilities of these genes from mTADA’s analyses. Columns ‘dn_MiD_CHD2018’ and ‘dn_LoF_CHD2018’ are data from an independent dataset. Column ‘pPoisson’ describes p values of the Poisson test for the independent dataset. Column ‘Known gene’ shows whether a gene is in the list of known genes (Yes/Y) or not (No/N). The last column shows p-values calculated by Jin et al.^23^ for their top significant genes.

*EE*. There were 16 genes. Similar to top CHD genes, their protein products also had more interactions than expected by using the STRING database (PPI *p* = 3e-11, Supplementary Fig. 6). Three genes *HECW2*, *MLL*, *WDR19* were not in the list of EE genes on the Online Mendelian Inheritance in Man^26^. These three genes only had PP < 0.3 in extTADA. Interestingly, *HECW2* had a DNM in a whole-genome-sequencing study recently^27^.

*SCZ*. There were 12 genes including *AUTS2*, *BRPF1*, *CHD8*, *HIST1H1E*, *HIVEP3*, *MAP4K4*, *MKI67*, *POGZ*, *SCN2A*, *SETD1A*, *SYNGAP1*, *TAF13*. These genes’ protein products were significantly connected by using the STRING database (PPI *p* = 1.6e-03, Supplementary Fig. 6). In these genes, only *TAF13* and *SETD1A* were suggested as top genes in previous studies^10,28^. In addition, *AUTS2* was reported as a SCZ gene from a common variant based study^29^. We tested these genes on an independent dataset of 4,877 cases and 6,203 controls, *HIST1H1E* showed nominally significant (Supplementary Table 3).

### Biological insights into shared and specific genes from mTADA’s analysis

**Fig. 6:**
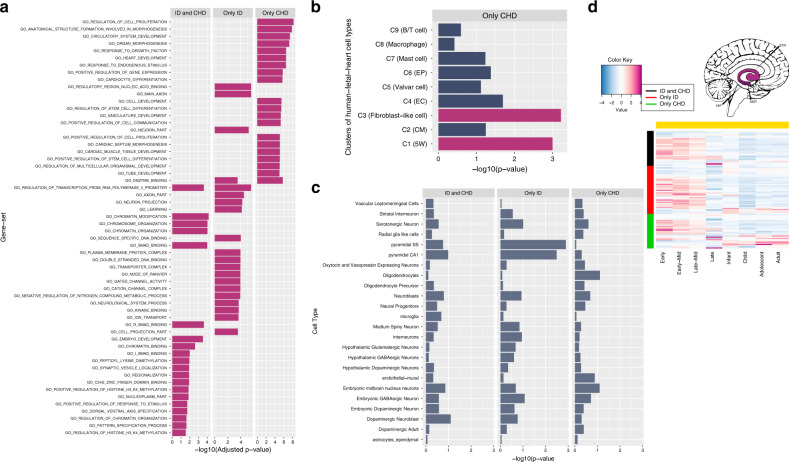
The analysis results of shared and specific gene lists for ID and CHD (Only CHD: CHD-specific genes, Only ID: ID-specific genes, ID and CHD: shared genes). **a** Top enrichment results of gene-ontology (G0) gene sets. These are the top 20 enriched gene sets of each gene list. All these results have adjusted-*p*-value < 0.05. **b** Enrichment results of human single-cell RNA sequencing (scRNAseq) datasets. These cells are from cardiac cells of the human fetal heart. They were clustered into 9 clusters (e.g., C1 to C9). The information of these clusters is in brackets (5W: 5-week hearts, ECs: endothelial cells, CMs: cardiomyocytes, epicardial cells: Eps). Magma-red bars are for results with adjusted *p*-value < 0.05 **c**) Enrichment results of mouse scRNAseq expression data. **d** BrainSpan expression results for the three gene lists. This is for Region 3 as defined by Huckins, et al.^34^ including hippocampus (HIP), amygdala (AMY), striatum (STR) regions. The package cerebroViz^67^ was used to draw brain regions.

To demonstrate the application of mTADA in helping to understand the shared and specific biological mechanism of two analyzed disorders. We extracted three gene lists (shared and specific genes) for each pair of disorders using a threshold of PP > 0.5. To increase the sample size for CHD, we combined both tested and independent datasets (Methods). We then focused on ID and CHD in this analysis because this pair of disorders had high numbers of risk genes for the three lists (30 shared genes, 40 ID-specific genes and 30 CHD-specific genes, Supplementary Data 2). Different systems biology approaches were used to test these three gene lists. First, we conducted gene-set enrichment analyses^30^ using gene-ontology (GO) gene sets^31^. The majority of top enriched GO gene sets were related to heart/cardiocyte-development for CHD-specific genes, to chromatin modification or DNA binding for shared or ID-specific genes (Fig. 6a). Next, we used gene sets from a human single-cell RNA sequencing (scRNAseq) dataset of ~4,000 cardiac cells from human embryos^32^. No overlaps were observed between shared or ID-specific genes with these gene sets, but interestingly the CHD-specific genes were enriched in multiple gene sets (Fig. 5b). We then tested the three gene lists by using mouse scRNAseq gene expression datasets from different brain regions^33^. The three gene lists were not significantly enriched in these cell types; however, for pyramidal cells, ID-specific genes were nominally significant while CHD-specific genes did not have the same direction (Fig. 5c). Finally, we used BrainSpan RNAseq gene expression data to cluster these three gene lists into spatiotemporal groups. Using eight time points and four regions as in recent studies^10,34^, shared and ID-specific genes were strongly expressed in the prenatal stages of the human brain while CHD-specific genes were expressed in both prenatal and postnatal stages for Region 3 including hippocampus, amygdala and striatum (Fig. 5d, Supplementary Fig. 7). The three other brain regions did not show strong differentiations between these three gene lists (Supplementary Fig. 7).

## Discussion

In this paper, we propose a method to jointly analyze two traits (mTADA) using de novo exome sequencing data. The method is an extension of our previous work for single traits^10,11^. mTADA estimates the proportion of overlapping risk genes (*π*_3_) between two traits, and then uses this information to infer how many overlapping risk genes exist between two traits. The pipeline is also able to infer the number of risk genes for each trait by calculating posterior probabilities (PPs) of genes for each trait. On simulated data, mTADA performs better than a single-trait approach, extTADA, on the identification of risk genes (Fig. 2). We applied mTADA to more than 13,000 trios of five neuropsychiatric disorders and congenital heart disease, and reported overlapping genes between these disorders. We also saw that mTADA reported more risk genes for these disorders than extTADA (Fig. 4). This suggests that mTADA can help in the identification of additional risk genes, especially for disorders whose large sample sizes are challenging to obtain or whose mean relative risks are small. For such disorders, users can combine the data of the disorders with large public data sets (e.g., trio data of ASD, DD) to prioritize risk genes. Using one-trait information to leverage the information for other traits has been successful in fine-mapping^35^ and common-variant^36^ studies. Based on our best knowledge, mTADA is the first tool using this approach for de novo mutation data. We hope that mTADA (https://github.com/hoangtn/mTADA) will be generally useful for analyzing de novo mutation data across complex traits.

By using mTADA for prioritizing top genes, multiple overlapping genes were observed for CHD, DD, ID and ASD. This replicates a recent study^37^ in which high overlapping genes were observed for CHD and neurodevelopmental disorders. Interestingly, CHD did not show any overlapping information with another neurodevelopmental disorder: EE. Two genes *SCN2A* and *POGZ* which have been reported as risk genes for some of these disorders^23,38–40^ are top overlapping genes from mTADA (Fig. 4d), but they show different trends. No *SCN2A* DNMs are in CHD data and no *POGZ* DNMs are in EE data. One possible reason is that the sample size of EE is small in this study (356 trios). Another hypothesis might be that they do not have strong overlapping biological pathways. We did not see any overlapping information between SCZ and CHD, or SCZ and EE. We analyzed in depth the top prioritized genes of CHD, EE and SCZ (Fig. 5, Supplementary Fig. 6). Some top risk CHD and EE genes from mTADA are also reported in recent studies^23,27^. Multiple top CHD genes have only one DNM, but have DNMs in independent data sets (Table 2). This suggests that they might be real risk-genes for this disorder. Interestingly, we identify 20 CHD genes (posterior probabilities >0.8) which are not in the list of 253 curated known human/mouse CHD genes. 3 of these 20 genes have DNMs in an independent data set. This shows the benefit of using mTADA in the prediction of risk genes for CHD by borrowing the information of DD (Fig. 5, Table 2). We used different systems biology approaches to understand the shared and specific risk gene lists of ID and CHD. Some specific information emerged from these analyses. CHD-specific genes were enriched for heart/cardiocyte pathways, cell types while shared and ID-specific genes were strongly expressed in the prenatal stages of the human brain and enriched in regulatory and binding pathways (Fig. 6). This suggests that a model-based approach as mTADA can help shed light on the shared and specific biological mechanism between disorders with larger sample sizes.

Although mTADA performs better than the single-trait based extTADA, it does have some limitations. mTADA uses the parameters of single traits from extTADA to infer *π*_3_. Using parameters from extTADA makes mTADA much faster in its calculation, it means mTADA relies on the results of the single-trait pipeline extTADA that uses a full Bayesian approach. Also, mTADA as well as extTADA use de novo counts for each gene and divide these counts into different categories similar to other rare variant based studies^12,41–43^. In this current pipeline, we estimated *π*_3_ directly from data. However, common-variant based genetic co-heritabilities^44^ and transcriptomic correlations^45^ for multiple pairs of NPDs are available now. Other studies which are able to incorporate the annotation information of each mutation, prior information for *π*_3_ from previous studies may increase the power of mTADA or similar tools. In the current version, users can set a prior or change a distribution for *π*_3_. Comorbid information might be used as prior information for *π*_3_ in the analyses of mTADA. For example, our estimated gene-level genetic overlaps which are inferred from *π*_3_’s estimations are very high for pairs of EE and ID (~31%, CI = 18.9–43.7%), ASD and ID (~38.1, CI = 30–46.6%). These three disorders are also highly comorbid^46,47^; therefore, this information may be used as priors. However, genomic results and comorbid information might not always have the same trends. For example, the genetic overlapping information between ASD and SCZ are high in our study (~16%, CI = 5.1–31.4%), in previous common-variant based (*r*_g_ = 0.16, se = 0.06, *p* = 0.0071) and transcriptomic studies (rho ~ 0.5, *p* < 0.001, Supplementary Table 2), but the comorbidity of the two disorders might not be strong or almost zero in some recent studies^48,49^. In addition, ASD and SCZ can have overlapping copy number variable regions^50,51^; however, duplications can be significantly seen for one disorder and deletions can highly present in the other disorder^51^. Finally, for all analyzed disorders, even though we observed multiple overlapping genes for pairs of disorders, the origin of these overlaps could be different. For example, for each pair, some overlapping genes could have more loss-of-function DNMs for one disorder and more missense damaging DNMs for the other disorder, and vice versa. Future rare-variant studies which are able to obtain comorbidity information from the overlapping samples and compare this with the genetic information will shed light on the genetic and clinical relationship of these disorders. Also, studies which are designed to understand in depth the information of variant categories for overlapping genes can elucidate the genomic mechanism of disorders.

Our analysis of de novo mutation data of neuropsychiatric disorders and CHD also has some limitations, in particular, overlapping phenotypes may lead to violation of mTADA assumptions. DD samples include people with different disorders^52^ and some of CHD samples may have other neuropsychiatric disorders^23^. In a recent study, the DD dataset was combined with the ID dataset to create a larger ID dataset because of the high proportion of people with ID inside the DD cohort^53^. In this study, even though we analyzed DD and ID separately to better understand the gene-level genetic overlaps between ID and other disorders, overlapping phenotypes may still affect the current results. We tested possible scenarios of overlapping phenotypes (Supplementary Note 1). The proportion of overlapping risk genes was modestly affected by ascertainment bias or by low percentages of misdiagnosed cases (<20%). However, this metric might be overestimated if misclassification rates were substantially high and the gene-level mean relative risks of one disorder were greatly different from those of the other disorder (Supplementary Note 1). The inflation could have an impact on analysis results, especially shared and specific risk genes. Nevertheless, mTADA always performed better than extTADA in the identification of risk genes for single-trait analyses in tested scenarios. It is possible that mTADA would benefit by jointly modeling these biases and this will be a future extension of the method.

With further development, the mTADA approach can be generalized further to consider more than two traits simultaneously, and the increased information could increase the number of identified risk genes but at a cost of increased computational time. Currently, the number of hypotheses increases exponentially to 2^N^ with N being the number of traits. To reduce computational time, another approach which uses a small number of latent probability vectors^54^ might be used for more than two-trait studies.

In conclusion, mTADA can be very useful for better understanding the genetic correlation across disorders (via the proportion of overlapping risk genes), and to prioritize additional risk genes for disorders. The approach of mTADA can be used to identify shared/specific risk genes for different categories of one trait (e.g., loss of function and missense de novo mutations). Genetic information of de novo mutations and rare case/control variants can be different^55^, mTADA might be adopted to pipelines which are able to apply to DNMs and rare case/control variants as two traits.

## Methods

### mTADA: statistical models and parameter estimation

The mTADA is designed to jointly analyze two traits using DNMs. We use statistical models of extTADA, a single-trait method, to model DNM counts for each trait in mTADA as presented in Table 1. The likelihood of the data across all *N* genes can be computed as $L = \mathop {\prod}\nolimits_{i = 1}^N {\mathop {\sum}\nolimits_{j = 0}^3 {\pi _j} } P_{ij}^1P_{ij}^2$ with $P_{ij}^k = P(x_{ki}|\phi _{kj})$, where *x*_*ki*_ and *ϕ*_*kj*_ are the *i*^*th*^ gene data and the *j*^*th*^ modelʼs parameters for trait *k* (*k* = 1, 2). In addition, if the data include multiple categories of variants then $P_{ij}^k = \mathop {\prod}\nolimits_{l = 1}^{n_C} {P_{ij}^{k_l}}$with *n*_*C*_ being the number of categories. For gene *i*, the statistical support for the *j*^*th*^ model is captured by its posterior probability ($PP_{ij} = \frac{{\pi _jP_{ij}^1P_{ij}^2}}{{\mathop {\sum }\nolimits_{m = 0}^3 \pi _mP_{im}^1P_{im}^2}}$, abbreviated as PP0, PP1, PP2 or PP3 for a gene).

We use our single-trait pipeline, extTADA, to estimate the proportions of risk genes ($\pi _1^S$ and $\pi _2^S$), mean relative risks ($\bar \gamma _1^S$ and $\bar \gamma _2^S$) and dispersion parameters ($\beta _1^S$ and $\beta _2^S$) for each single trait (described as the superscript). We use these values inside mTADA: $\pi _1 = \pi _1^S - \pi _3$, $\pi _2 = \pi _2^S - \pi _3$, and *π*_0_ = 1 − ($\pi _1^S + \pi _2^S - \pi _3$) because of $\mathop {\sum}\nolimits_{j = 0}^3 {\pi _j} = 1$. We assume that $\bar \gamma _1 = \bar \gamma _1^S$; $\bar \gamma _2 = \bar \gamma _2^S$; $\beta _1 = \beta _1^S$ and $\beta _2 = \beta _2^S$. Therefore, we only estimate *π*_3_ inside mTADA. Bayesian models are built using the *rstan* package^56^. We use Markov Chain Monte Carlo (MCMC) within *rstan* to estimate *π*_3_. Convergence is diagnosed by the estimated potential scale reduction statistic ($\hat R$) and visualizing traces of results. The *Locfit* package^57^ is used to obtain the mode, CI of *π*_3_. We use the mode as the estimated value of *π*_3_. We also tested a model with different mean relative risks for shared and specific risk genes. The model was more complex for the estimation process of parameters but did not improve the risk-gene identification. Therefore, this complex model was not used in our analysis.

### Generation and analyses of simulated data

We simulated DNMs for genes under the mTADA model in Table 1. All 19,538 genes and their mutation rates from our current real dataset were used. A gene was assigned to one of the four groups (four models) by using the probability (*π*_0_, *π*_1_, *π*_2_, *π*_3_). We used ${\uppi}_1^{\mathrm{S}} = 0.05$ and ${\uppi}_2^{\mathrm{S}} = 0.03$ which are approximately equal to ASD, ID and DD results in our single-trait study^10^. π_3_ was simulated with different values between 0 to min(${\uppi}_1^{\mathrm{S}},\,{\uppi}_2^{\mathrm{S}}$); and *π*_0_, *π*_1_ and *π*_2_ were calculated as described in the section above. A range of mean relative risks were simulated for each of the two traits. Two mutation categories were simulated for each trait; therefore, there were four mean relative risks for the two traits. We used results from our previous studies^10,11^ and other studies^58,59^ for simulated values of mean relative risks. We simulated 100 values of each combination of π_3_ and mean relative risks. We then calculated the mean of these 100 simulation results.

To calculate the proportion of false positive genes when there was not a genetic overlap between two tested disorders, we simulated different combinations of genetic parameters with *π*_3_ = 0. For each PP threshold, we divided the number of identified overlapping genes by the total tested genes (*n* = 19,358 genes in our analysis).

We also used simulated data to assess the correlation between true and observed *π*_3_ values and between PPs and oFDRs. An oFDR at a PP threshold was defined as the number of false positive genes divided by the number of identified genes. To use mTADA for single traits, for the *i*^*th*^ gene, we calculated *PP*_*i*1_ + *PP*_*i*3_ and *PP*_*i*2_ + *PP*_*i*3_ for the first and second trait respectively.

To compare risk gene classification performance between mTADA and extTADA on single traits, we used AUCs. We calculated true and false positive rates for extTADA and mTADA across PP thresholds, and calculated the areas under these ROC curves.

### Real datasets of de novo mutations and variants

For primary analyses, we used the DNM data collected by Nguyen et al.^10^ and CHD data from Homsy et al.^16^. These data included 356 EE trios; 5,122 ASD trios; 4,293 DD trios; 1,012 ID trios; 1,077 SCZ trios; and 1,213 CHD trios. DNMs were annotated and classified into multiple categories as in our previous work^10^ as follows. For EE, ASD, DD, ID, and CHD, we used two categories^10^: loss-of-function (LoF) and missense damaging (MiD) DNMs. The LoF category included nonsense, essential splice site, and frameshift DNMs defined by Plink/Seq^60^ while the MiD category included DNMs annotated as missense by Plink/Seq and predicted damaging by each of seven methods^41^: SIFT, Polyphen2_HDIV, Polyphen2_HVAR, LRT, PROVEAN, MutationTaster, and MutationAssessor. For SCZ, we used LoF, MiD and synonymous mutations within DNase I hypersensitive sites because this category showed significant DNM enrichment in SCZ probands^22^ and non-null mean relative risk in extTADA^10^. Mutation rates were calculated as described by Fromer, et al.^60^ and Nguyen, et al.^10^.

For the validation of mTADA’s results and for better understanding the specific and shared risk genes between tested disorders, other datasets were used in the analysis. First, we used independent datasets to validate mTADA results. For CHD, we extracted variant data of 2,871 probands from Jin et al.^23^. These samples include 2,445 trios (1,204 trios are inside the data set of extTADA and used in the primary analysis of this study) and 226 singletons^23^. Only independent CHD samples were used in the validation process. For EE, we used the whole-genome-sequencing trio data of Hamdan et al.^27^. This dataset includes 197 trios not included in our mTADA analyses. For SCZ, a case/control independent SCZ dataset from Genovese et al.^41^ was used. Disruptive and damaging ultra-rare variants from 4,877 cases and 6,203 controls were extracted from Table S3 of the study^41^.

### Known risk-gene datasets

We extracted lists of known risk genes from two sources. 253 curated known human/mouse CHD genes were obtained from the supplementary data set 2 of Jin et al.^23^. A list of EE genes from the Online Mendelian Inheritance in Man^26^ was downloaded on September 02, 2019 using keywords “epileptic encephalopathy” and “epileptic encephalopathies”.

### Gene expression datasets

Human scRNAseq expression datasets of 4,000 cardiac cells were from 18 human embryos which ranged from 5 weeks (5W) to 25W of gestation. These were classified into four major cell types (cardiomyocytes (CMs), cardiac fibroblasts, endothelial cells (ECs), and valvar interstitial cells (VICs)), and also filtered and clustered into nice clusters. Gene lists of the nine clusters were extracted from Table S2 of Cui et al.^32^. scRNAseq transcriptome datasets were obtained from Skene et al.^33^ via the link: http://www.hjerling-leffler-lab.org/data/scz_singlecell/ (Downloaded on August 01, 2018). These datasets included 9,970 single cells. These cells were clustered into 24 different cell types. Spatiotemporal transcriptome data were obtained from BrainSpan^61^, divided into eight developmental time points (four prenatal and four postnatal)^62^. The average expression at each spatiotemporal point was calculated across samples. For each gene, average expression values were standardized across spatiotemporal points to obtain z-scores^10,34^. Z-scores were used for visualizing gene lists.

### Analysis of de novo mutations using mTADA

extTADA was used to obtain the proportions of risk genes and the mean relative risks of each category for each disorder. These values were then used as input for mTADA to estimate π_3_ and then to calculate PP_ij_ $({\mathrm{i}} = 1..{\mathrm{N}},\,{\mathrm{j}} = 0..3,\,{\mathrm{N}} = 19,358\,{\mathrm{genes}})$ for each pair of traits. The default algorithm, No-U-Turn Sampler (NUTS), in the rstan package was used to estimate π_3_. Two independent chains and 10,000 steps for each chain were used in the sampling process. Only 1,000 samples from each chain were chosen for further analyses.

For primary analysis, we applied mTADA to NPDs and 1,213 CHD trios. For understanding the specific and shared risk genes between tested disorders, we combined both tested and independent datasets of CHD (2,445 trios) in jointly analyzing with other disorders to increase power. Finally, we also applied mTADA to the two CHD datasets (tested and independent datasets) to test the performance of the method as described in Supplementary Note 2.

### Other statistical methods for real data analyses

We used the EWCE package^63^ to calculate the enrichment of our gene lists and the expression data from the 24 mouse cell types. To test the significance of the overlap of two gene sets, a permutation approach was used. We chose two random gene sets whose lengths are the same as the two tested gene sets from the background genes (19,358 genes from mTADA). This was carried out *N* times (*N* = 10,000 in this study) and the numbers of overlapping genes were recorded in a vector *m*. A p-value was calculated as $(length\left( {m\left[ {m > m_0} \right]} \right) + 1){\mathrm{/}}(length\left( m \right) + 1)$) in which *m*_0_ is the observed number of overlapping genes between the two tested gene sets. To conduct PPI analyses, we used the STRING database and the package STRINGdb^25^ from the Bioconductor project^64^, and p-values of protein-protein interactions were extracted from these analyses. To examine expression information of identified genes, we used the package mclust^65^ to cluster BrainSpan gene expression data (z-scores) in heatmap analyses. To test the significance for individual genes from DNMs, we used a Poisson test. The R function $ppois(y - 1,\,lambda = 2 \times Ntrio \times \mu , \,lower.tail = FALSE)$ in which *y* and *μ* are the number of DNMs and the mutation rate of the tested gene; *Ntrio* is the number of trios. All analyses were carried out using the R software^66^.

### Reporting summary

Further information on research design is available in the Nature Research Reporting Summary linked to this article.

## Supplementary information

Supplementary Information

Supplementary Data 1

Supplementary Data 2

Description of Additional Supplementary Files

Reporting Summary

## Data Availability

All analyzed results are in Supplementary Data 1 and 2. These supplementary datasets are also available at: https://github.com/hoangtn/mTADA.

## Code availability

mTADA package and examples for reproducible studies are available online (https://github.com/hoangtn/mTADA).

## References

[1] Solovieff N, Cotsapas C, Lee PH, Purcell SM, Smoller JW (2013). Pleiotropy in complex traits: challenges and strategies. Nat. Rev. Genet..

[2] Zhernakova A (2011). Meta-analysis of genome-wide association studies in celiac disease and rheumatoid arthritis identifies fourteen non-HLA shared loci. PLoS Genet..

[3] Galesloot TE, van Steen K, Kiemeney LA, Janss LL, Vermeulen SH (2014). A comparison of multivariate genome-wide association methods. PLoS ONE.

[4] Allison DB (1998). Multiple phenotype modeling in gene-mapping studies of quantitative traits: power advantages. Am. J. Hum. Genet..

[5] Pickrell JK (2016). Detection and interpretation of shared genetic influences on 42 human traits. Nat. Genet..

[6] Giambartolomei C (2014). Bayesian test for colocalisation between pairs of genetic association studies using summary statistics. PLoS Genet..

[7] Turley, P. et al. Multi-trait analysis of genome-wide association summary statistics using MTAG. *Nat. Genet.* **50**, 229–237 (2018). 10.1038/s41588-017-0009-4 PMC5805593 29292387

[8] Lutz SM, Fingerlin TE, Hokanson JE, Lange C (2017). A general approach to testing for pleiotropy with rare and common variants. Genet. Epidemiol..

[9] Guo, B. & Wu, B. Integrate multiple traits to detect novel trait-gene association using GWAS summary data with an adaptive test approach. *Bioinformatics* **35**, 2251–2257 (2019). 10.1093/bioinformatics/bty961 PMC6596889 30476000

[10] Nguyen HT (2017). Integrated Bayesian analysis of rare exonic variants to identify risk genes for schizophrenia and neurodevelopmental disorders. Genome Med..

[11] He X (2013). Integrated model of de novo and inherited genetic variants yields greater power to identify risk genes. PLoS Genet..

[12] De Rubeis S (2014). Synaptic, transcriptional and chromatin genes disrupted in autism. Nature.

[13] Iossifov I (2014). The contribution of de novo coding mutations to autism spectrum disorder. Nature.

[14] Li J (2016). Genes with de novo mutations are shared by four neuropsychiatric disorders discovered from NPdenovo database. Mol. Psychiatry.

[15] Hoischen A, Krumm N, Eichler EE (2014). Prioritization of neurodevelopmental disease genes by discovery of new mutations. Nat. Neurosci..

[16] Homsy J (2015). De novo mutations in congenital heart disease with neurodevelopmental and other congenital anomalies. Science.

[17] Willsey AJ (2018). The psychiatric cell map initiative: a convergent systems biological approach to illuminating key molecular pathways in neuropsychiatric disorders. Cell.

[18] Lelieveld SH (2016). Meta-analysis of 2,104 trios provides support for 10 new genes for intellectual disability. Nat. Neurosci..

[19] Deciphering Developmental Disorders Study. (2017). Prevalence and architecture of de novo mutations in developmental disorders. Nature.

[20] Wang S (2018). De novo sequence and copy number variants are strongly associated with tourette disorder and implicate cell polarity in pathogenesis. Cell Rep..

[21] Cappi, C. et al. De novo damaging DNA coding mutations are associated with obsessive-compulsive disorder and overlap with touretteʼs disorder and autism. *Biol. Psychiatry* 10.1016/j.biopsych.2019.09.029 (2019). 10.1016/j.biopsych.2019.09.029 PMC7160031 31771860

[22] Takata A, Ionita-Laza I, Gogos JA, Xu B, Karayiorgou M (2016). De novo synonymous mutations in regulatory elements contribute to the genetic etiology of autism and schizophrenia. Neuron.

[23] Jin SC (2017). Contribution of rare inherited and de novo variants in 2,871 congenital heart disease probands. Nat. Genet.

[24] Heyne HO (2018). De novo variants in neurodevelopmental disorders with epilepsy. Nat. Genet.

[25] Szklarczyk D (2017). The STRING database in 2017: quality-controlled protein-protein association networks, made broadly accessible. Nucleic Acids Res..

[26] Amberger JS, Bocchini CA, Scott AF, Hamosh A (2019). OMIM.org: leveraging knowledge across phenotype-gene relationships. Nucleic Acids Res..

[27] Hamdan FF (2017). High rate of recurrent de novo mutations in developmental and epileptic encephalopathies. Am. J. Hum. Genet..

[28] Fromer M (2012). Discovery and statistical genotyping of copy-number variation from whole-exome sequencing depth. Am. J. Hum. Genet..

[29] Zhang B (2014). Association study identifying a new susceptibility gene (AUTS2) for schizophrenia. Int J. Mol. Sci..

[30] Subramanian A (2005). Gene set enrichment analysis: a knowledge-based approach for interpreting genome-wide expression profiles. Proc. Natl Acad. Sci. USA.

[31] Ashburner M (2000). Gene ontology: tool for the unification of biology. The Gene Ontology Consortium. Nat. Genet..

[32] Cui Y (2019). Single-cell transcriptome analysis maps the developmental track of the human heart. Cell Rep..

[33] Skene NG (2018). Genetic identification of brain cell types underlying schizophrenia. Nat. Genet.

[34] Huckins LM (2019). Gene expression imputation across multiple brain regions provides insights into schizophrenia risk. Nat. Genet..

[35] Kichaev G (2017). Improved methods for multi-trait fine mapping of pleiotropic risk loci. Bioinformatics.

[36] Maier RM (2018). Improving genetic prediction by leveraging genetic correlations among human diseases and traits. Nat. Commun..

[37] Ware JS, Samocha KE, Homsy J, Daly MJ (2015). Interpreting de novo variation in human disease using denovolyzeR. Curr. Protoc. Hum. Genet..

[38] White J (2016). POGZ truncating alleles cause syndromic intellectual disability. Genome Med..

[39] Stessman HAF (2016). Disruption of POGZ is associated with intellectual disability and autism spectrum disorders. Am. J. Hum. Genet..

[40] Ben-Shalom R (2017). Opposing effects on NaV1.2 function underlie differences between SCN2A variants observed in individuals with autism spectrum disorder or infantile seizures. Biol. Psychiatry.

[41] Genovese G (2016). Increased burden of ultra-rare protein-altering variants among 4,877 individuals with schizophrenia. Nat. Neurosci..

[42] Allen AS (2017). Ultra-rare genetic variation in common epilepsies: a case-control sequencing study. Lancet Neurol..

[43] Sanders SJ (2012). De novo mutations revealed by whole-exome sequencing are strongly associated with autism. Nature.

[44] Cross-Disorder Group of the Psychiatric Genomics Consortium. (2013). Genetic relationship between five psychiatric disorders estimated from genome-wide SNPs. Nat. Genet..

[45] Gandal MJ (2018). Shared molecular neuropathology across major psychiatric disorders parallels polygenic overlap. Science.

[46] Amiet C (2008). Epilepsy in autism is associated with intellectual disability and gender: evidence from a meta-analysis. Biol. Psychiatry.

[47] Tuchman R, Cuccaro M (2011). Epilepsy and autism: neurodevelopmental perspective. Curr. Neurol. Neurosci. Rep..

[48] Volkmar FR, Cohen DJ (1991). Comorbid association of autism and schizophrenia. Am. J. Psychiatry.

[49] Leyfer OT (2006). Comorbid psychiatric disorders in children with autism: interview development and rates of disorders. J. Autism Dev. Disord..

[50] Kushima I (2018). Comparative analyses of copy-number variation in autism spectrum disorder and schizophrenia reveal etiological overlap and biological insights. Cell Rep..

[51] Crespi B, Stead P, Elliot M (2010). Evolution in health and medicine Sackler colloquium: comparative genomics of autism and schizophrenia. Proc. Natl Acad. Sci. USA.

[52] DDD Study. (2017). Prevalence and architecture of de novo mutations in developmental disorders. Nature.

[53] Taylor JL (2019). Paternal-age-related de novo mutations and risk for five disorders. Nat. Commun..

[54] Wei Y, Tenzen T, Ji H (2015). Joint analysis of differential gene expression in multiple studies using correlation motifs. Biostatistics.

[55] Sifrim A (2016). Distinct genetic architectures for syndromic and nonsyndromic congenital heart defects identified by exome sequencing. Nat. Genet..

[56] Carpenter B (2016). Stan: a probabilistic programming language. J. Stat. Softw..

[57] Loader, C. Locfit: local regression, likelihood and density estimation. *R package version* **1** (2007).

[58] Willsey AJ (2017). De novo coding variants are strongly associated with tourette disorder. Neuron.

[59] Epi K. Consortium. (2013). De novo mutations in epileptic encephalopathies. Nature.

[60] Fromer M (2014). De novo mutations in schizophrenia implicate synaptic networks. Nature.

[61] Miller JA (2014). Transcriptional landscape of the prenatal human brain. Nature.

[62] Lin GN (2015). Spatiotemporal 16p11.2 protein network implicates cortical late mid-fetal brain development and KCTD13-Cul3-RhoA pathway in psychiatric diseases. Neuron.

[63] Skene NG, Grant SG (2016). Identification of vulnerable cell types in major brain disorders using single cell transcriptomes and expression weighted cell type enrichment. Front. Neurosci..

[64] Gentleman RC (2004). Bioconductor: open software development for computational biology and bioinformatics. Genome Biol..

[65] Scrucca L, Fop M, Murphy TB, Raftery AE (2016). mclust 5: clustering, classification and density estimation using Gaussian finite mixture models. R. J..

[66] R Core Team. R: A Language and Environment for Statistical Computing. (Vienna, Austria, 2018).

[67] Bahl E, Koomar T, Michaelson JJ (2017). cerebroViz: an R package for anatomical visualization of spatiotemporal brain data. Bioinformatics.

