# Supplementary material for: mTADA is a framework for identifying risk genes from de novo mutations in multiple traits
Source: Nat Commun. 2020 Jun 10;11:2929. doi: 10.1038/s41467-020-16487-z (PMC7287090; doi:10.1038/s41467-020-16487-z)
Supplement: Supplementary file 1 — Supplementary Information [file 41467_2020_16487_MOESM1_ESM.pdf]

mTADA is a framework for identifying risk genes from de novo mutations in multiple traits

Nguyen et al., 2020

Supplementary Information

Table of Contents

Supplementary notes 2

Supplementary Note 1: Simulation studies of the effects of misdiagnosis and ascertainment bias on analysis results. 2

Results 2

Methods 3

Supplementary Note 2: Real data analysis for two datasets of one trait. 5

Supplementary Tables 6

Supplementary Table 1 6

Supplementary Table 2 7

Supplementary Table 3 8

Supplementary Table 4 9

Supplementary Figures 10

Supplementary Figure 1 10

Supplementary Figure 2 11

Supplementary Figure 3 12

Supplementary Figure 4 13

Supplementary Figure 5 14

Supplementary Figure 6 15

Supplementary Figure 7 16

Supplementary Figure 8 17

Supplementary Figure 9 18

Supplementary Figure 10 19

Supplementary Figure 11A 20

Supplementary Figure 11B. 21

Supplementary Figure 12 22

Supplementary Figure 13 23

Supplementary Figure 14 24

Supplementary Figure 15A 25

Supplementary Figure 15B 26

Supplementary Figure 16 27
Reference:..*28*

Supplementary notes

Supplementary Note 1: Simulation studies of the effects of misdiagnosis and ascertainment bias on analysis results.

Results

For samples from neuropsychiatric disorders, a proportion of individuals from Trait 1 group might be from Trait 2 group and vice versa, due to disease misdiagnosis. In another scenario of ascertainment bias, individuals with both disorders may be over-sampled into one of the two groups. These situations might inflate estimates of the proportion of shared risk genes. We designed simulation studies to test whether these scenarios could substantially impact on the estimates of π_3 and on the power of risk-gene identification. These scenarios could happen for both groups (bi-direction). To simplify the simulation process, we focused on the simulation setting where only data of Trait 1 is subject to misdiagnosis or ascertainment bias. However, similar approaches could be used for Trait 2 or both traits. For each combination of parameters, 100 replicates were carried out.

Misdiagnosed cases. We first assessed the impact of sample misdiagnosis on mTADA parameter estimation and risk gene discovery. We assumed that a percentage of samples from Trait 1 group were misdiagnosed, i.e. they were actually from Trait 2 group as in Supplementary Table 4. We simulated several possible percentages that likely occur in the real data (1%, 5% and 10%). In addition, a high percentage (20%) was simulated to test the impact of extreme misdiagnosis. Overall, when this value was less than 20%, the inflation of π_3 was not high if the two tested traits had similar mean relative risks (Supplementary Figure 8). Estimated values of π_3 were not affected by misdiagnosed cases if the mean relative risks of Trait 2 group were much smaller than those of Trait 1 group (e.g., 105/29 for the mean relative risks of Trait 1 and 12/2 for those of Trait 2). However, if the mean relative risks of Trait 2 group were substantially higher than those of Trait 1 group, π_3's estimates converged to the risk-gene proportion of Trait 2 (π_2^S) when the percentage of misdiagnosed cases was very high (~ 20%, Supplementary Figure 8). This was because π_2^S was smaller than π_1^S (the risk-gene proportion of Trait 1) in our simulations (Method Section). Second, we tested the correlations between estimated and simulated values of π_3. This proportion was not affected when the percentage of misdiagnosed cases was less than 20%. However, it was underestimated when the percentage was large (20%, Supplementary Figure 9). Next, we tested in depth the classification results of single traits when there were misdiagnosed cases. We compared AUCs between the single-trait approach (extTADA) and mTADA (Supplementary Figure 10). mTADA performed better than extTADA in almost all situations, especially when π_3 increased. As expected, the performance of mTADA was much better for Trait 2 group because this group had samples in Trait 1 group. Finally, we tested the observed FDRs (oFDRs) of each trait from mTADA's results (Supplementary Figure 11). For Trait 2 group,

oFDRs were well calibrated (Supplementary Figure 11B). For Trait 1 group, oFDRs were also similar to those of Trait 1 group when the percentages of misdiagnosed cases were not high (<20%) or Trait-2's mean relative risks were much smaller than Trait-1's. However, oFDRs were inflated for high misdiagnosis situations (20% in Supplementary Figure 11A). The highest inflation rates were observed for small values of π_3 (0.01 or 0.015). Such situations might not be seen in real data because $\pi_3's$ values and the percentage of misdiagnosis should be highly correlated.

Ascertainment bias. In Trait 1 samples, some cases will happen to have the Trait 2 phenotype, because of the comorbidity of the diseases. When the samples with comorbidity condition get over-sampled – ascertainment bias, this leads to a higher number of individuals with Trait 2 in the Trait 1 samples, than otherwise (no ascertainment bias). As a result, genes involved in Trait 2, i.e. Trait 2 specific risk genes and shared risk genes, may have higher DNM counts than otherwise. We denote R, the ratio of inflated DNM counts and the DNM counts in the absence of ascertainment bias. A large value of R potentially leads to biased estimates of model parameters, in particular π_3. We derived an expression of R, as a function of comorbidity and the extent of ascertainment bias in Supplementary Methods. We then simulated results under possible values of R from 1.1 to 1.7.

There was not inflation for π_3 when R was <1.5. When R was ≥ 1.5, slightly overestimates were observed for this measurement (Supplementary Figure 12). As a result, π_1 was not strongly affected by this bias (Supplementary Figure 13). mTADA also performed better than extTADA in the risk-gene classification: mTADA's AUCs were higher than extTADA's AUCs (Supplementary Figure 14). Finally, oFDRs were well calibrated with PPs for both groups (Supplementary Figure 15A and B).

Methods

Simulation studies

Misdiagnosed cases. We assumed that a percentage of Trait 1 group was from Trait 2 group; therefore, gene-level DNMs of these samples followed the same statistical models as those of Trait 2 samples. Details of the simulation process are described in Supplementary Table 4.

Ascertainment bias. We formulated an expression to calculate the inflation ratio, R, of gene-level DNMs when there was ascertainment bias. Our goal is to estimate the expected number of mutations of a gene in the data of Trait 1, when there is ascertainment bias. Consider one sample at a time: let X be the mutation status of the gene of interest (1 if the gene has a mutation and 0 otherwise); and Y_1, Y_2 be the phenotype status of two traits. Let sample size of trait 1 be N_1 and π be the proportion of samples with both traits in Trait 1 group. The expected number of mutations in the gene is given by:

$$E = N_1 \pi P(X = 1 | Y_1 = 1, Y_2 = 1) + N_1(1 - \pi) P(X = 1 | Y_1 = 1, Y_2 = 0) \quad (1)$$

where the first term comes from comorbid samples and the second from samples with only trait 1. When the two traits are unrelated, we should have $P(X = 1 | Y_1 = 1, Y_2 = 1) = P(X = 1 | Y_1 = 1)$,

which reduces to the standard mTADA model. However, when there is comorbidity, this equality may not hold.

We first estimate $P(X=1|Y_1=1, Y_2=1)$. In particular, we are interested in comparing this with $P(X=1|Y_1=1)$. If the ratio is substantially higher than 1, it means that in samples with both phenotypes, the rate of mutation would be higher, comparing with samples with $Y_1=1$ only. Using Bayes rule, we write $P(X=1|Y_1=1, Y_2=1)$ as:

$$P(X=1|Y_1=1, Y_2=1) = \frac{P(Y_1=1, Y_2=1, X=1)}{P(Y_1=1, Y_2=1)} = P(X=1)\frac{P(Y_1=1|X=1)}{P(Y_1=1)}\frac{P(Y_2=1|Y_1=1, X=1)}{P(Y_2=1|Y_1=1)} \quad (2)$$

Because the product of the first two terms is just $P(X=1|Y_1=1)$; therefore, we have this ratio:

$$\frac{P(X=1|Y_1=1, Y_2=1)}{P(X=1|Y_1=1)} = \frac{P(Y_2=1|Y_1=1, X=1)}{P(Y_2=1|Y_1=1)} = \lambda \quad (3)$$

We note this ratio depends on the risk gene status. When the gene is not a risk gene of Trait 2 (H_0 or H_1), λ should be 1. When the gene is a risk gene of Trait 2 (H_2 or H_3), the ratio may be higher than 1. This will be discussed later.

To compute $P(X=1|Y_1=1, Y_2=0)$, we introduce an additional parameter:

$$P(Y_2=1|Y_1=1) = c; \quad P(Y_2=0|Y_1=1) = 1-c \quad (4)$$

The parameter c captures the extent of comorbidity of two disorders in the general population. Note that c and π (actual proportion in our Trait 1 samples) may differ, reflecting ascertainment bias. We use the Bayes rule:

$$\frac{P(X=1|Y_1=1, Y_2=0)}{P(X=1|Y_1=1)} = \frac{P(Y_2=0|Y_1=1, X=1)}{P(Y_2=0|Y_1=1)} = \frac{1-P(Y_2=1|Y=1, X=1)}{P(Y_2=0|Y_1=1)} = \frac{1-\lambda c}{1-c} \quad (5)$$

Now, we have the expected number of mutations in Trait 1 data:

$$E = N_1 P(X=1|Y_1=1)[\pi\lambda + (1-\pi)\frac{1-\lambda c}{1-c}] \quad (6)$$

We can now compute the expected mutations in a gene, depending on which hypothesis (H_0 to H_3) applies to the gene. We denote μ the mutation rate of a gene per chromosome copy.

. H_0: $\lambda = 1$, and $P(X=1|Y_1=1) = 2\mu$ therefore, $E = N_1 2\mu$.

. H_1: $\lambda = 1$, and $P(X=1|Y_1=1) = 2\gamma_1\mu$; therefore, $E = N_1 2\gamma_1\mu$.

. H_2: λ may be high than 1, and $P(X=1|Y_1=1) = 2\mu$; therefore,

$$E = N_1 2\mu[\pi\lambda + (1-\pi)\frac{1-\lambda c}{1-c}] \quad (7)$$

. H_3: λ may be high than 1, and $P(X=1|Y_1=1) = 2\gamma_1\mu$; therefore,

$$E = N_1 2\gamma_1\mu[\pi\lambda + (1-\pi)\frac{1-\lambda c}{1-c}] \quad (8)$$

From equations (7) and (8), if there is no ascertainment bias (no over- or under-sampling), i.e. $\pi = c$, the expected rate would not change. When $\lambda > 1$, it is easy to see that E is a monotonic increasing function of π (given c and λ). The ratio of E and expected rate is the inflation ratio, R, as defined previously.

The value of R thus depends on λ. Intuitively, λ should be somewhat smaller than γ_2 (the effect of a risk gene on Trait 2). Suppose the co-morbidity is high, and it is mostly driven by inherited variants, then $P(Y_2 = 1|Y_1 = 1)$ is likely high, say 0.5, and having an extra mutation will not make this ratio significantly larger: λ would be at most 1/0.5 = 2. It is possible to estimate the value of λ using the liability threshold model. We assume $Y_1 = 1$ (mostly due to inherited variants) and $X = 1$ acts on the risk of $Y_2 = 1$ independently: at the liability scale, this means that $X = 1$ and $Y_2 = 1$ increase the liability in an additive fashion. With this assumption, one can derive the value of λ as a function of the prevalence of Trait 2 (which determines liability threshold), c, and relative risk γ_2. We do not pursue this derivation here, however. Instead, for our purpose, we only point out that under all settings, we always have $\lambda \leq 1/c$.

Assuming a pessimistic scenario of $\lambda = 1/c$, we can estimate the value of R. When c is low, it means that the two phenotypes are quite different, so ascertainment bias is likely low. When c is large, ascertainment bias can be higher. We consider some settings:

. $c = 0.1, \pi = 0.15$: $R = 1.15$.

. $c = 0.3, \pi = 0.5$: $R = 1.67$.

In the simulation process, we tested different R values between 1.1 and 1.7.

Supplementary Note 2: Real data analysis for two datasets of one trait.

We attempted to use mTADA to jointly analyze two CHD datasets as two independent disorders (CHD1 and CHD2) to test whether mTADA could be able to report a high proportion of overlapping risk genes for the two datasets, and to compare the performance of mTADA and extTADA.We applied mTADA and extTADA to two CHD datasets. extTADA was run separately for the main CHD dataset (CHD1, n = 1213 trios), the independent CHD dataset (CHD2, n = 1241 trios). mTADA was run for CHD1-CHD2. extTADA was used to estimate π_1^S and π_2^S. These two proportions were 0.0419 and 0.0485 for CHD1 and CHD2 respectively. Estimated by mTADA, π_3 was 0.0252 (credible interval, CI = 0.0136, 0.0388), which was 60.1% (CI=32.5-92.6%) of π_1^S and 52% (CI=28-80%) of π_2^S. mTADA performed better than extTADA in the identification of risk genes for single disorders by using overlapping information to leverage the statistical evidence of genes (Supplementary Figure 16).

Supplementary Tables

Disorder1	Disorder2	π_1^S	π_2^S	π_3	l_π_3	u_{π_3}	gO (%)	l_gO (%)	u_gO (%)
ASD	DD	0.04427	0.02936	0.01802	0.01472	0.02171	32.4	26.5	39
ASD	ID	0.04427	0.02708	0.01968	0.0155	0.02409	38.1	30	46.6
ASD	EE	0.04427	0.01548	0.00967	0.00525	0.01452	19.3	10.5	29
ASD	CHD	0.04427	0.0419	0.0174	0.00947	0.02748	25.3	13.8	40
DD	ID	0.02936	0.02708	0.02056	0.0176	0.02353	57.3	49.1	65.6
DD	EE	0.02936	0.01548	0.0065	0.00382	0.01007	17	10	26.3
DD	CHD	0.02936	0.0419	0.01891	0.01271	0.02618	36.1	24.3	50
ID	EE	0.02708	0.01548	0.01007	0.00614	0.01419	31	18.9	43.7
ID	CHD	0.02708	0.0419	0.01567	0.00881	0.02362	29.4	16.5	44.3
EE	CHD	0.01548	0.0419	0.00111	0	0.00704	2	0	12.5
SCZ	ASD	0.03237	0.04427	0.01056	0.00373	0.02076	16	5.6	31.4
SCZ	DD	0.03237	0.02936	0.00789	0.00355	0.01449	14.7	6.6	26.9
SCZ	ID	0.03237	0.02708	0.00419	0.00052	0.01094	7.6	0.9	19.8
SCZ	EE	0.03237	0.01548	0.00212	0	0.00979	4.6	0	21.4
SCZ	CHD	0.03237	0.0419	0.00275	0	0.0154	3.8	0	21.5

Supplementary Table 1

Risk-gene overlaps. π_1^S and π_2^S are the proportions of risk genes for single traits estimated by extTADA (Nguyen, et al., 2017). π_3 is the overlapping proportion of risk genes while l_π_3 and u_π_3 are the lower and upper values of π_3's credible intervals. The genetic overlap (gO) is calculated as in the main text or $gO = 100\% * \pi_3/(\pi_1^S + \pi_2^S - \pi_3)$.

Study	Result
Cross-Disorder Group of the Psychiatric Genomics Consortium, et al. (2013)	r_g = 0.16, se = 0.06, p = 0.0071
Cross-Disorder Group of the Psychiatric Genomics Consortium (2019)	r_g = 0.22, se = 0.04, 1.42e-05
Brainstorm Consortium, et al. (2018)	r_g = 0.2082, correlation se = 0.0577, p < 3.35e-04
Gandal, et al. (2018)	rho ~ 0.5, p < 0.001
Hammerschlag, et al. (2019)	r_g = 0.24, se = 0.039, p=1.09e-09

Supplementary Table 2

Genetic overlaps and transcriptomic correlation between ASD and SCZ in previous studies*. r_g: genetic correlation, se: standard error.*

geneName	dn_silentCFPK_SCZ	dn_damaging_SCZ	dn_lof_SCZ	dn_damaging_DD	CaseCount	ControlCount	pValue	pBH
AUTS2	0	0	1	0	3	1	0.191	0.44
BRPF1	0	0	1	0	6	5	0.185	0.44
CHD8	0	0	1	0	1	2	0.414	0.44
HIST1H1E	0	0	1	0	4	0	0.038	0.413
HIVEP3	0	0	1	0	2	1	0.325	0.44
MAP4K4	0	0	1	3	2	5	0.224	0.44
MKI67	0	0	2	0	1	6	0.095	0.44
POGZ	0	0	1	0	NA	NA	NA	NA
SCN2A	0	0	1	9	2	2	0.364	0.44
SETD1A	1	0	3	0	4	4	0.258	0.44
SYNGAP1	0	0	1	0	2	1	0.325	0.44
TAF13	0	0	2	0	1	0	0.44	0.44

Supplementary Table 3

Top SCZ risk genes from the joint analysis of SCZ and developmental disorders (DD). *Columns which start with 'dn' (de novo) describe the number of de novo mutations (DNMs) for each variant category, and these DNMs were used in the analysis of mTADA. Columns 'CaseCount' and 'ControlCount' show the counts of disruptive and damaging ultra-rare variants for cases and controls respectively. These case/control data are from an independent dataset of 4,877 cases and 6,203 controls.*

Information	Proportion	First trait		Second trait
**Trio number**		N_1		N_2
		These samples are correctly assigned to this group	These samples are misdiagnosed to this group	All samples are correctly assigned to this group
**Trio number in the simulation process**		$N_1(1-PE)$	N_1PE	N_2
$\boldsymbol{H_0}$	π_0	$x_{i1}^1 \sim Poisson(2N_1(1-PE))$	$x_{i1}^2 \sim Poisson(2N_1PE\mu_i)$	$x_{i2} \sim Poisson(2N_2PE\mu_i)$
$\boldsymbol{H_1}$	π_1	$x_{i1}^1 \sim Poisson(2N_1(1-PE)\mu_i\gamma_{i1})$<br>$\gamma_{i1} \sim Gamma(\bar{\gamma}_1\beta_1, \beta_1)$	$x_{i1}^2 \sim Poisson(2N_1PE\mu_i)$	$x_{i2} \sim Poisson(2N_2PE\mu_i)$
$\boldsymbol{H_2}$	π_2	$x_{i1}^1 \sim Poisson(2N_1(1-PE))$	$x_{i1}^2 \sim Poisson(2N_1PE\mu_i\gamma_{i2})$<br>$\gamma_{i2} \sim Gamma(\bar{\gamma}_2\beta_2, \beta_2)$	$x_{i2} \sim Poisson(2N_2PE\mu_i\gamma_{i2})$<br>$\gamma_{i2} \sim Gamma(\bar{\gamma}_2\beta_2, \beta_2)$
$\boldsymbol{H_3}$	π_3	$x_{i1}^1 \sim Poisson(2N_1(1-PE)\mu_i\gamma_{i1})$<br>$\gamma_{i1} \sim Gamma(\bar{\gamma}_1\beta_1, \beta_1)$	$x_{i1}^2 \sim Poisson(2N_1PE\mu_i\gamma_{i2})$<br>$\gamma_{i2} \sim Gamma(\bar{\gamma}_2\beta_2, \beta_2)$	$x_{i2} \sim Poisson(2N_2PE\mu_i\gamma_{i2})$<br>$\gamma_{i2} \sim Gamma(\bar{\gamma}_2\beta_2, \beta_2)$
**Gene-level variant count**		$x_{i1} = x_{i1}^1 + x_{i1}^2$		$x_{i2} = x_{i2}$

Supplementary Table 4

The simulation process of misdiagnosed cases for one variant category at the $\mathbf{i^{th}}$ gene. *There are N_1 samples in the first-trait group, and N_2 samples in the second-trait group. Assuming that a percentage (PE) of samples in the first-trait group* ***is from the second-trait group****; therefore, their variant counts at the gene are from the same distribution as the second-trait group.*

Supplementary Figures

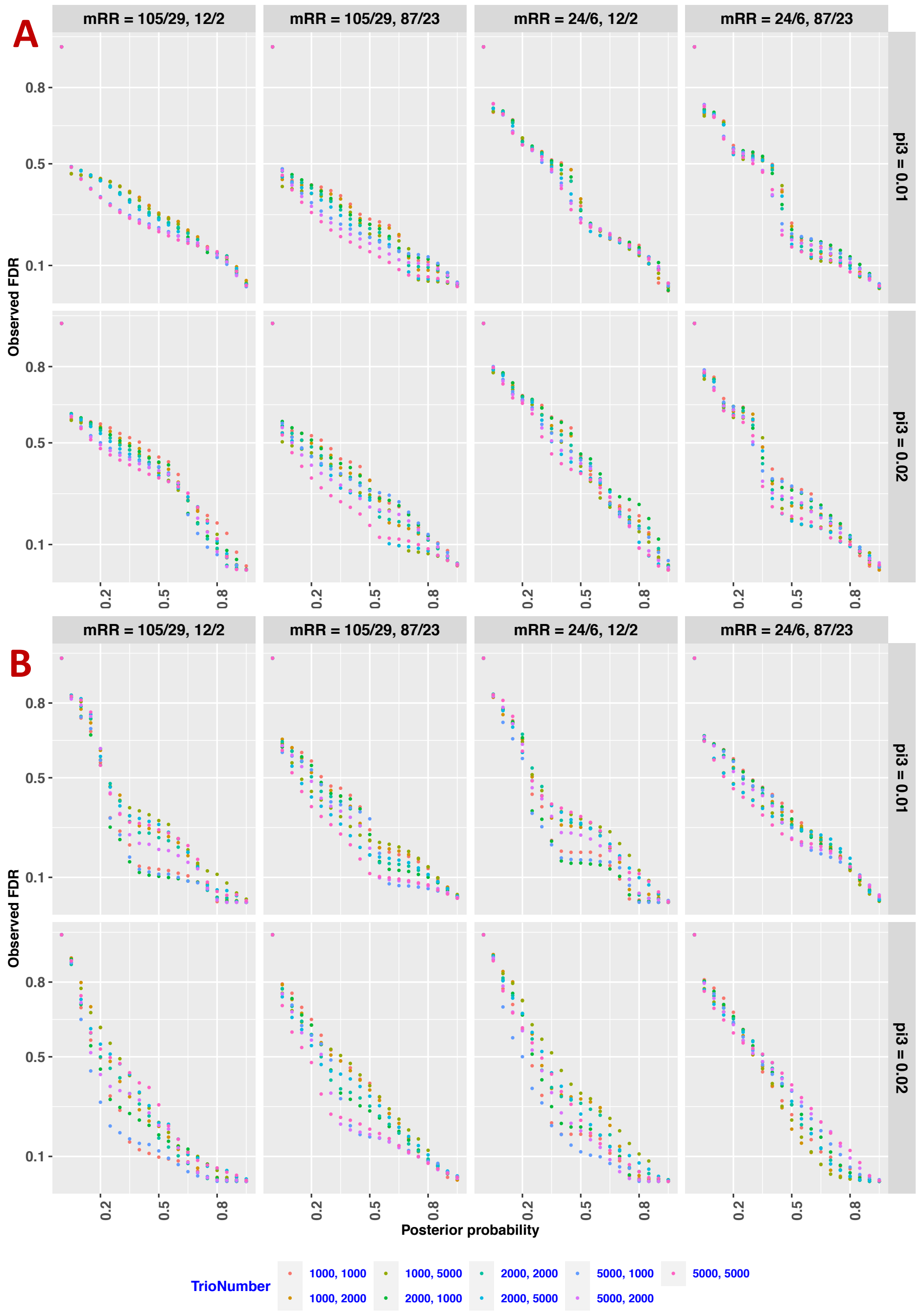

Supplementary Figure 1

Correlation between posterior probabilities (PPs) and observed false discovery rates (FDRs)*. The A and B panels show results for the two models: genes are associated with only the first trait (Model 1) and only the second trait (Model 2) in Figure 1 respectively. The PPs of these results are PP1 or PP2 in the main text.*

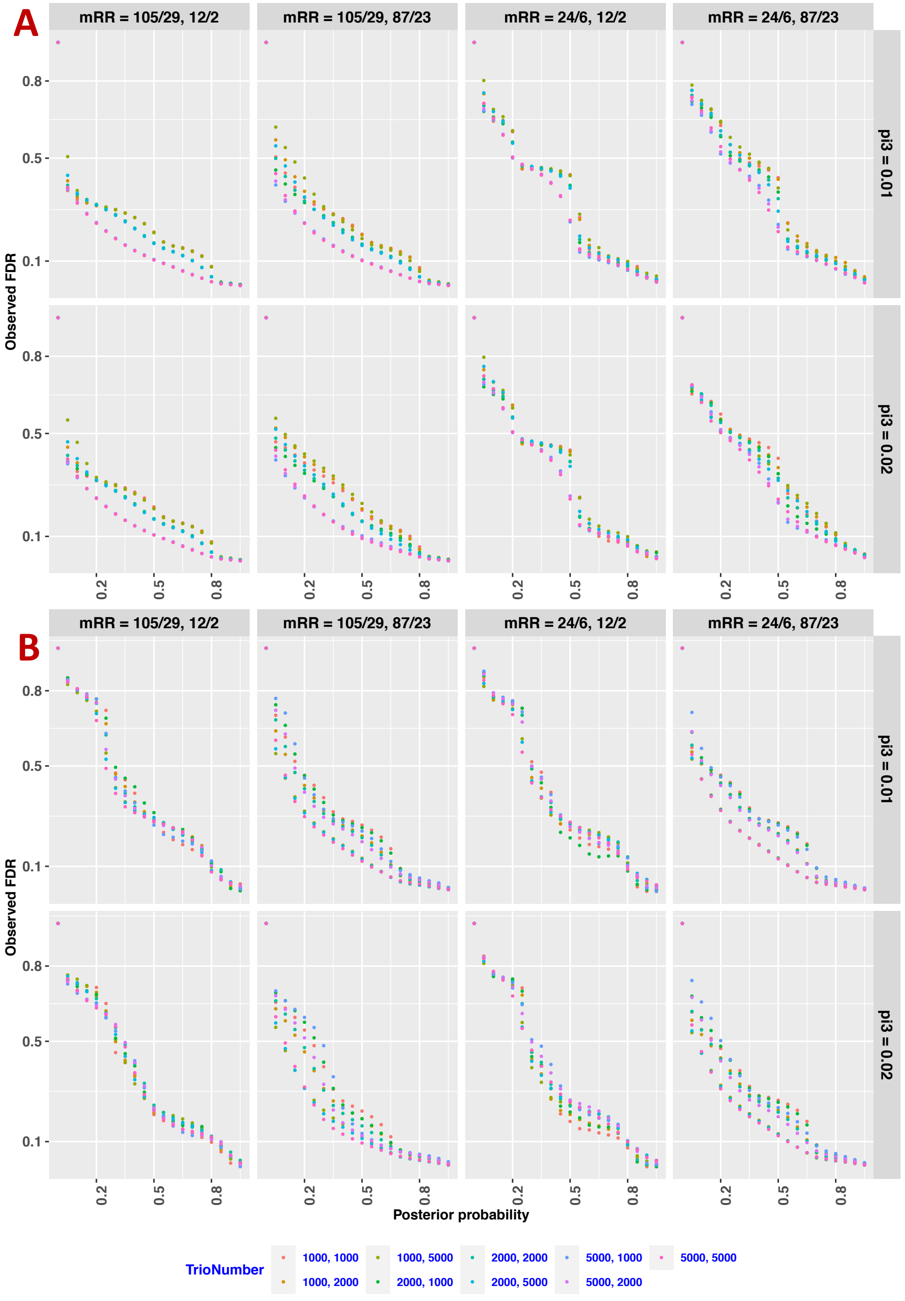

Supplementary Figure 2

***Correlation between posterior probabilities (PPs) and observed false discovery rates (FDRs)**. The A and B panels show results for the two traits: genes are associated with the first trait (Model 1 + 3) and the second trait (Model 2+3) respectively. The PPs of these results are PP1 + PP3 and PP1 + PP2 for the first and the second traits respectively.*

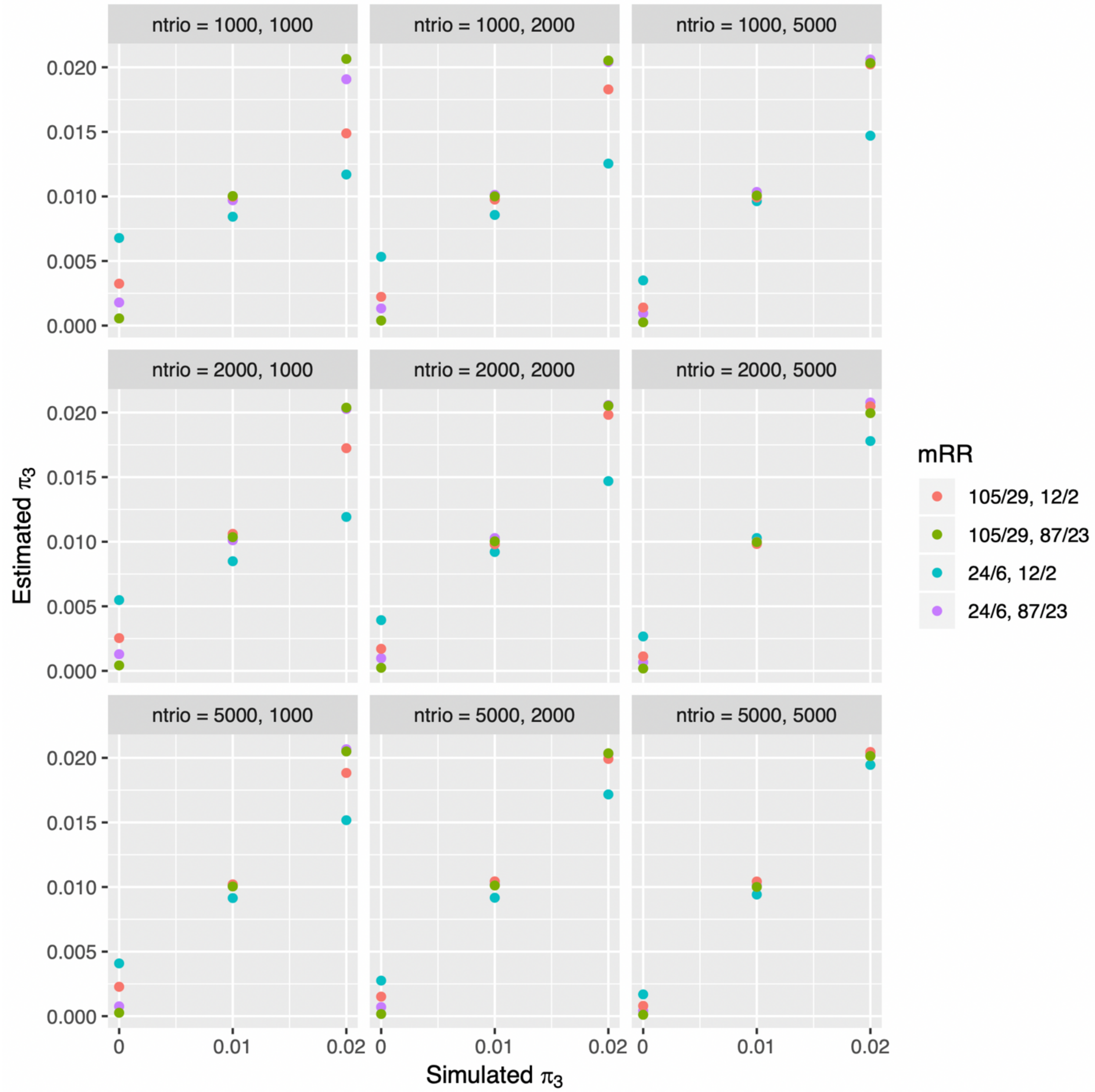

Supplementary Figure 3

Correlation between simulated and estimated values of the overlapping proportion of risk genes (π_3) for different trio sample sizes. *mRR: mean relative risks; ntrio: trio numbers of two traits.*

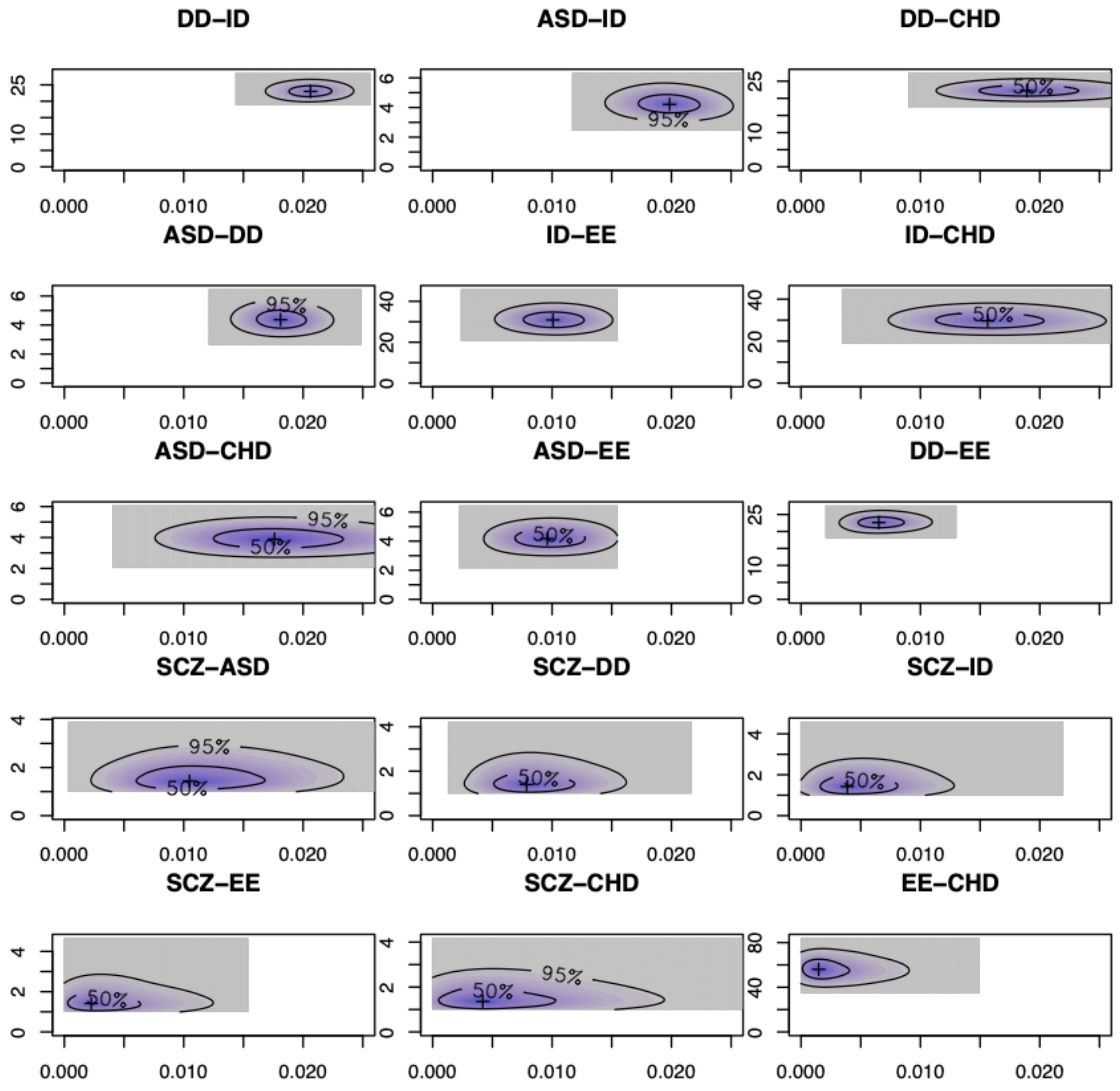

Supplementary Figure 4

Overlapping proportions of risk genes of disorders from Markov Chain Monte Carlo sampling results. *X-axes are for the overlapping proportions of risk genes while y axes represent the mean relative risks of the first traits.*

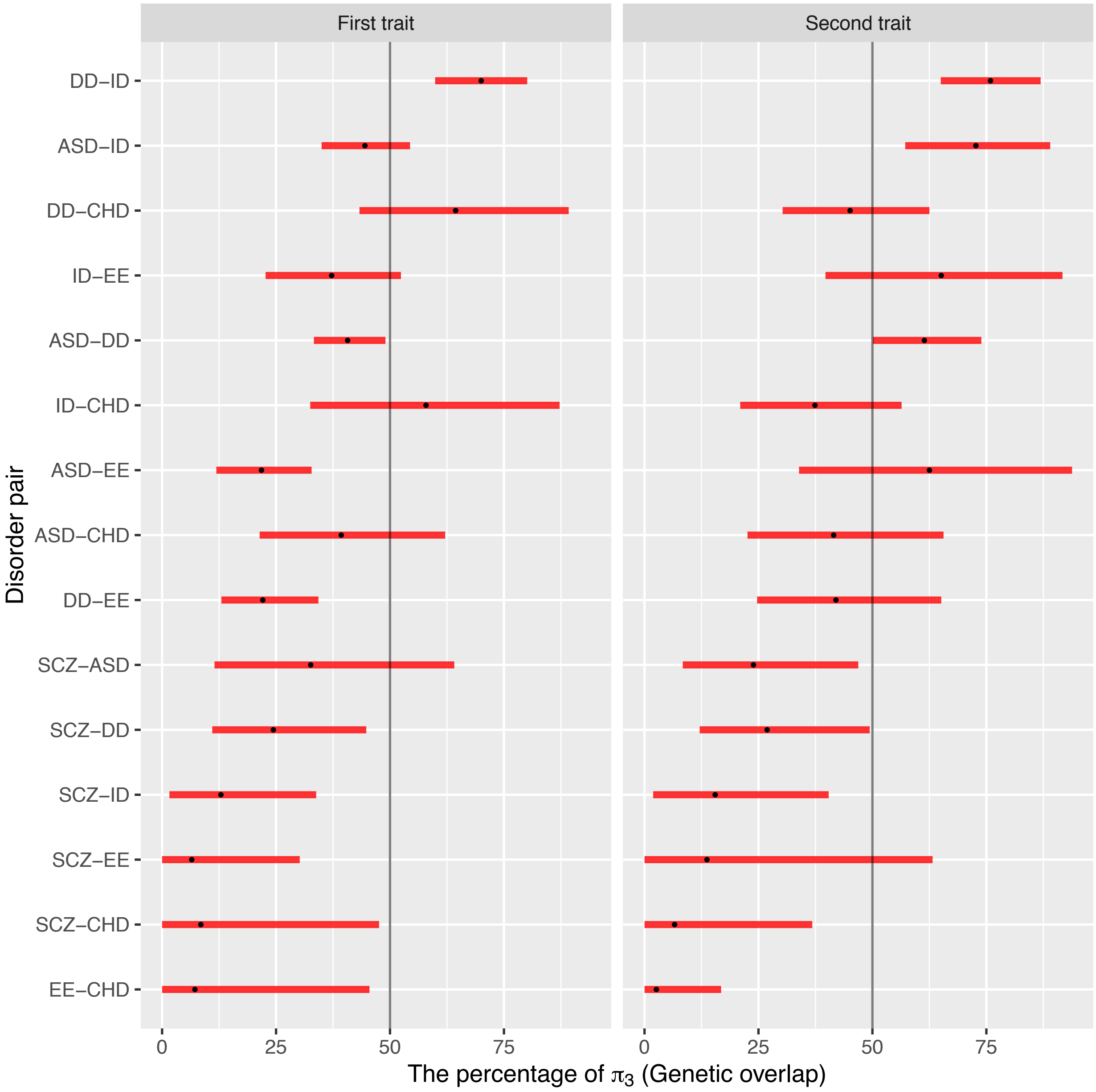

Supplementary Figure 5

The percentage of overlapping risk genes for single traits. *These values were calculated as* $100\pi_3/(\pi_1+\pi_3)$ *and* $100\pi_3/(\pi_1+\pi_3)$ *for the first and the second traits respectively.*

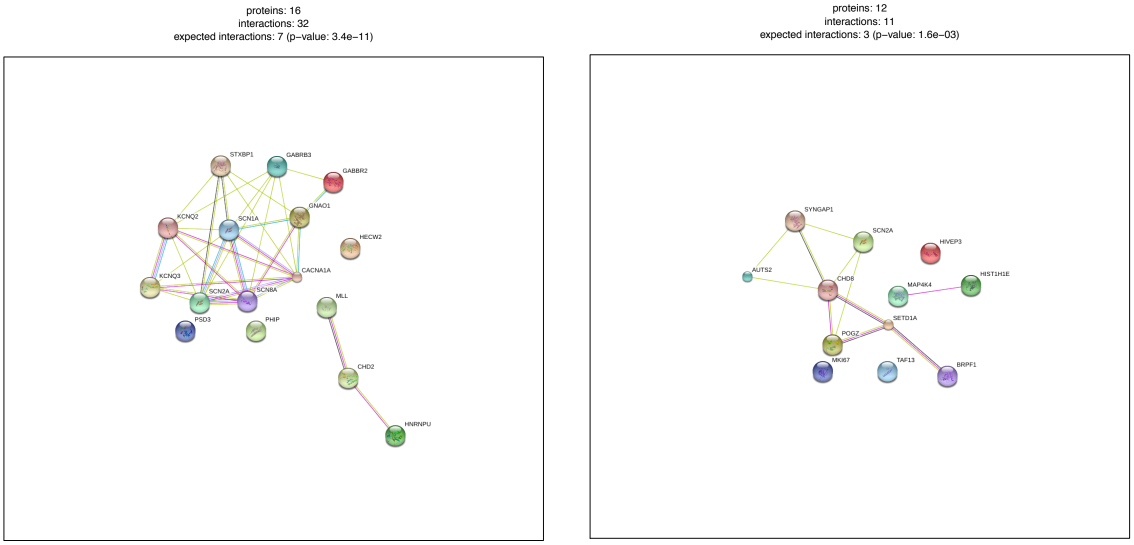

Supplementary Figure 6

Protein-protein interaction (PPI) results for top EE and SCZ genes (posterior probability>0.8).

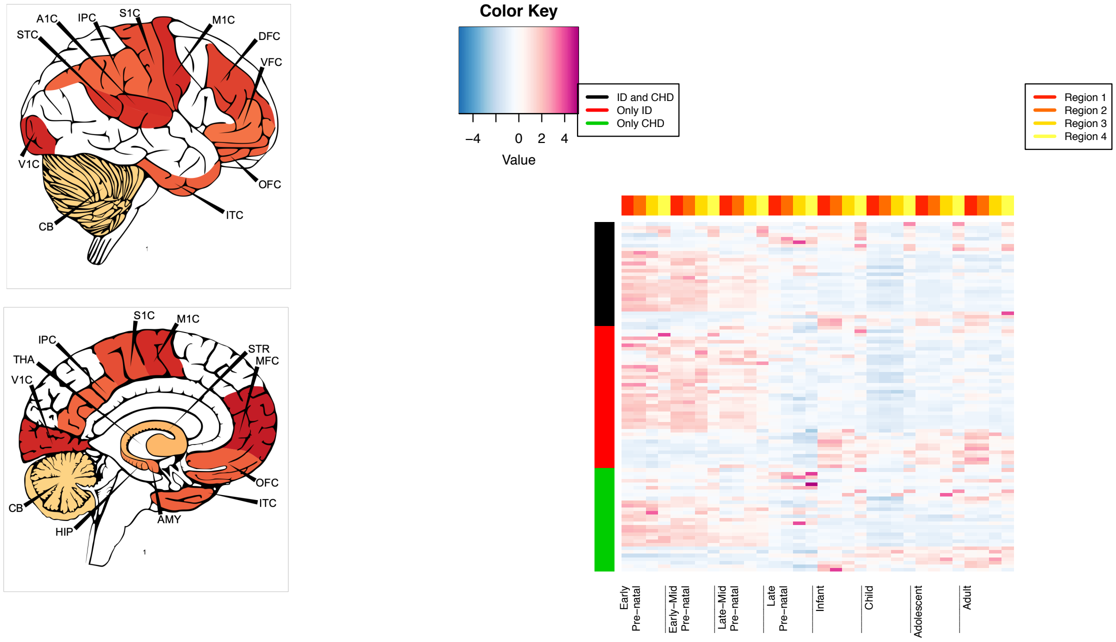

Supplementary Figure 7

Spatiotemporal expression results of top genes from the analysis of ID and CHD data across four brain regions. *Left pictures show different subregions, and the four regions are from these sub-regions (Region 1: IPC, V1C, ITC, OFC, STC, A1C; Region 2:S1C, M1C, DFC, VFC, MFC; Region 3:HIP, AMY, STR; Region 4: CB). The package cerebroViz (Bahl, et al., 2017) was used to draw brain regions. The abbreviation of these regions as described in the package: inferior parietal cortex (IPC), primary visual cortex (V1C), inferior temporal cortex (ITC), orbital frontal cortex (OFC), posterior (caudal) superior temporal cortex (STC), primary auditory cortex (A1C), primary somatosensory cortex (S1C), primary motor cortex (M1C), dorsolateral prefrontal cortex (DFC), ventrolateral prefrontal cortex (VFC), medial prefrontal cortex (MFC), hippocampus (HIP), amygdala (AMY), striatum (STR), cerebellum (CB).*

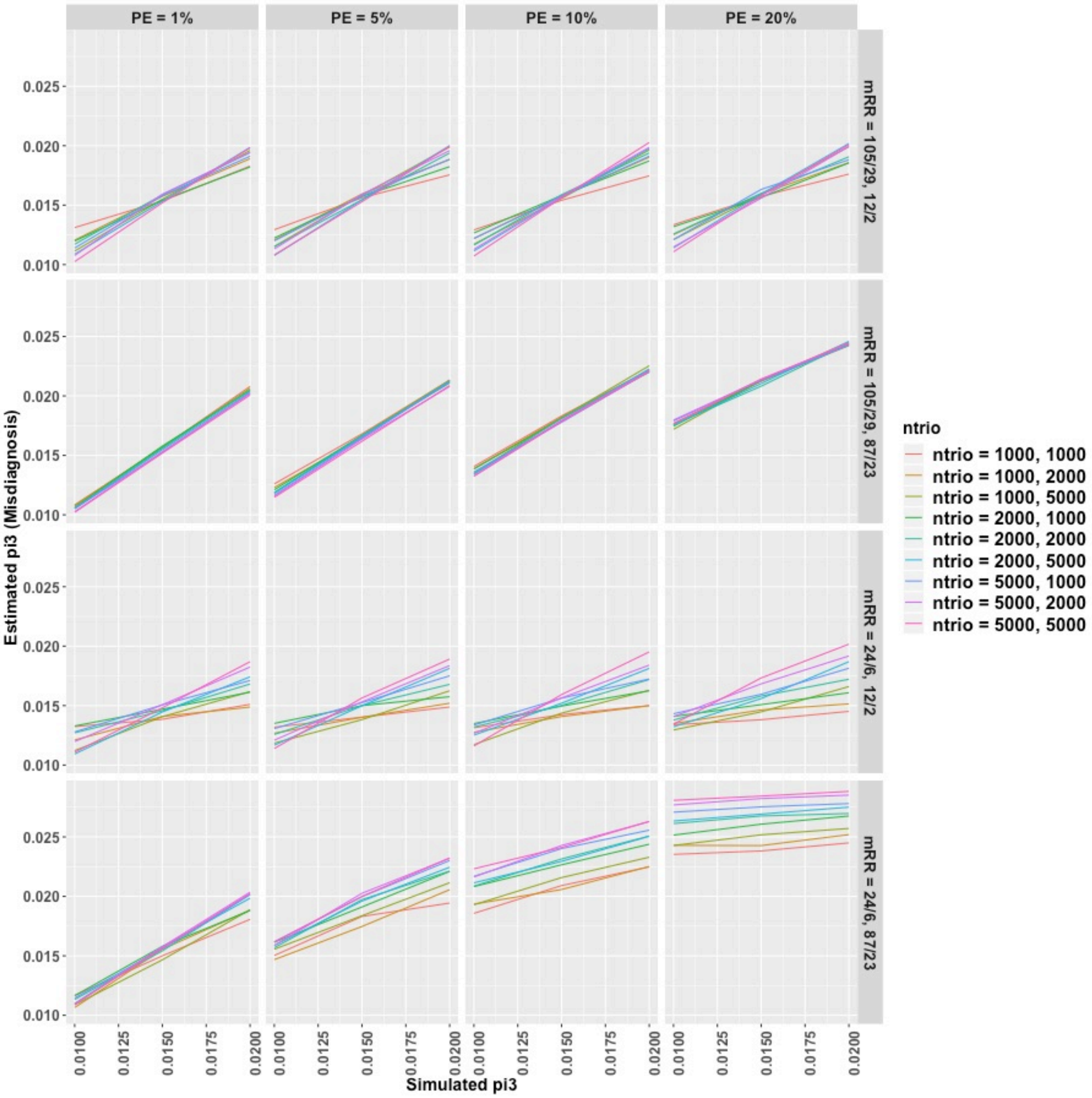

Supplementary Figure 8

The influence of misdiagnosed cases on the estimation of π_3. *X-axes show the simulated values of π_3 while Y-axes describe the estimated values of π_3. PE: the percentage of samples inside the first-trait group have the second trait (e.g., the first column shows that 1% of samples in the first-trait group are misdiagnosed, they belong to the second-trait group); mRR: mean relative risks; nTrio: the number of parent-offspring trios.*

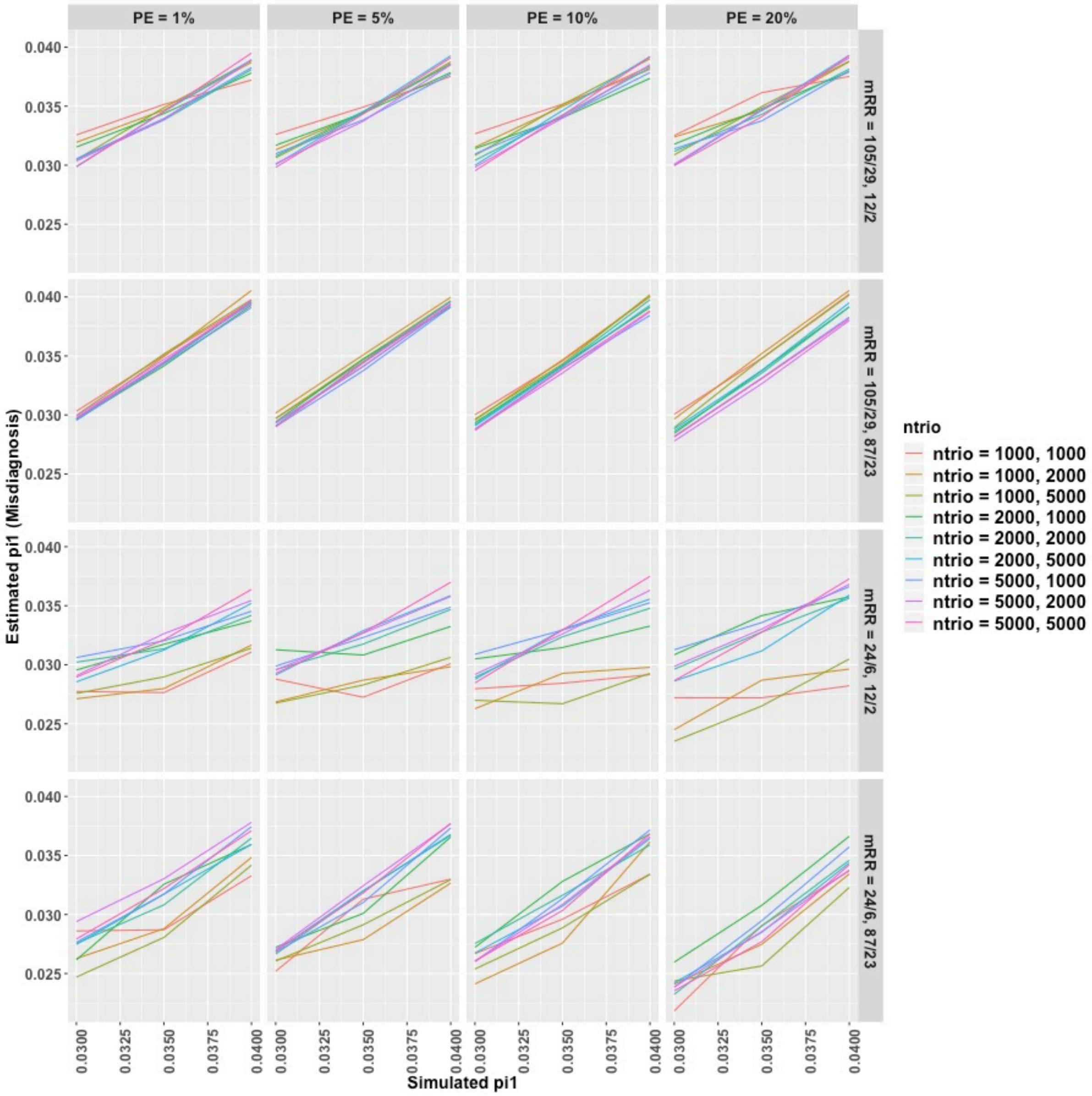

Supplementary Figure 9

The influence of misdiagnosed cases on the estimation of $\boldsymbol{\pi_1}$. *X-axes show the simulated values of π_1 while Y-axes describe the estimated values of π_1. PE: the percentage of samples inside the first-trait group which have the second trait (e.g., the first column shows that 1% of samples in the first-trait group are misdiagnosed, they belong to the second-trait group); mRR: mean relative risks; nTrio: the number of parent-offspring trios.*

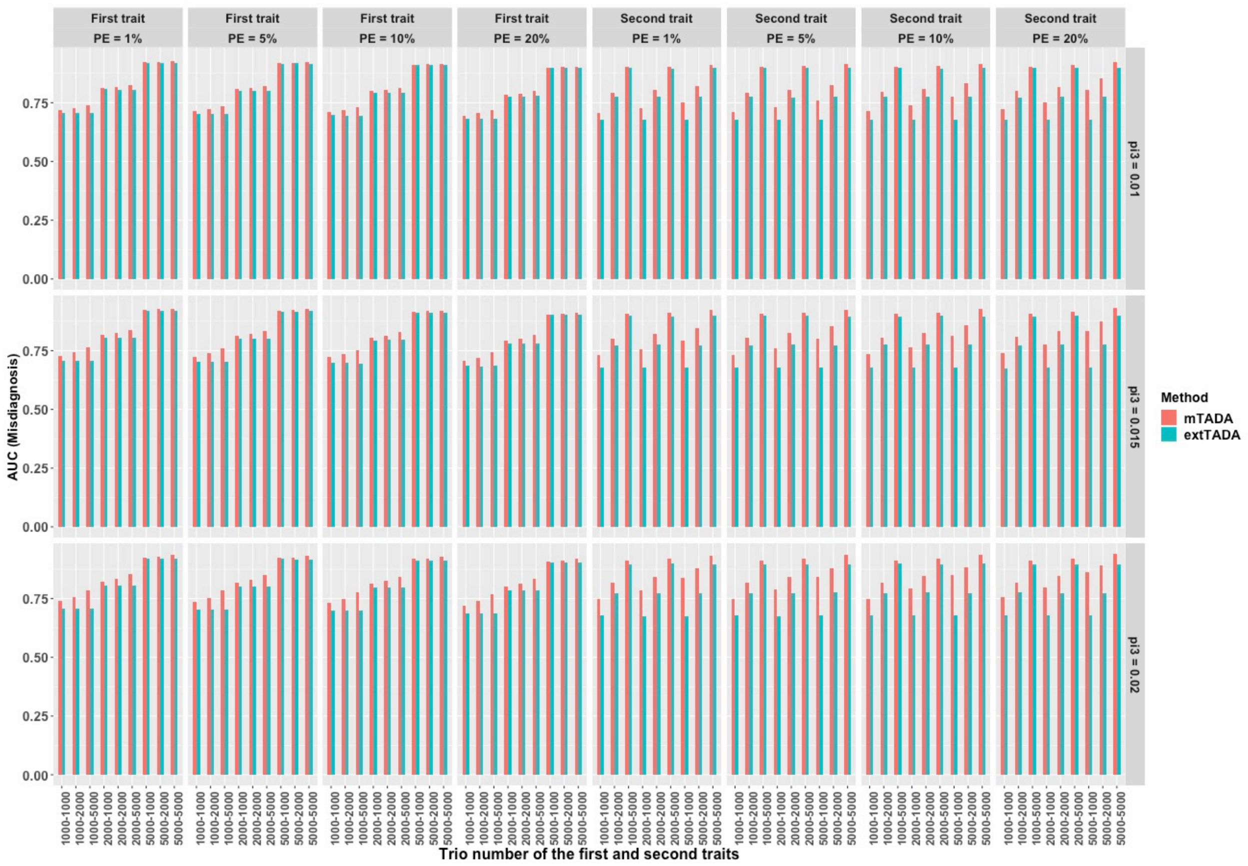

Supplementary Figure 10

Comparison of single-trait and two-trait methods when there are misdiagnosed cases. *X-axes show the number of parent-offspring trios while Y-axes describe AUC values. PE: the percentage of samples inside the first-trait group which have the second trait (e.g., the first column shows that 1% of samples in the first-trait group are misdiagnosed, they belong to the second-trait group).*

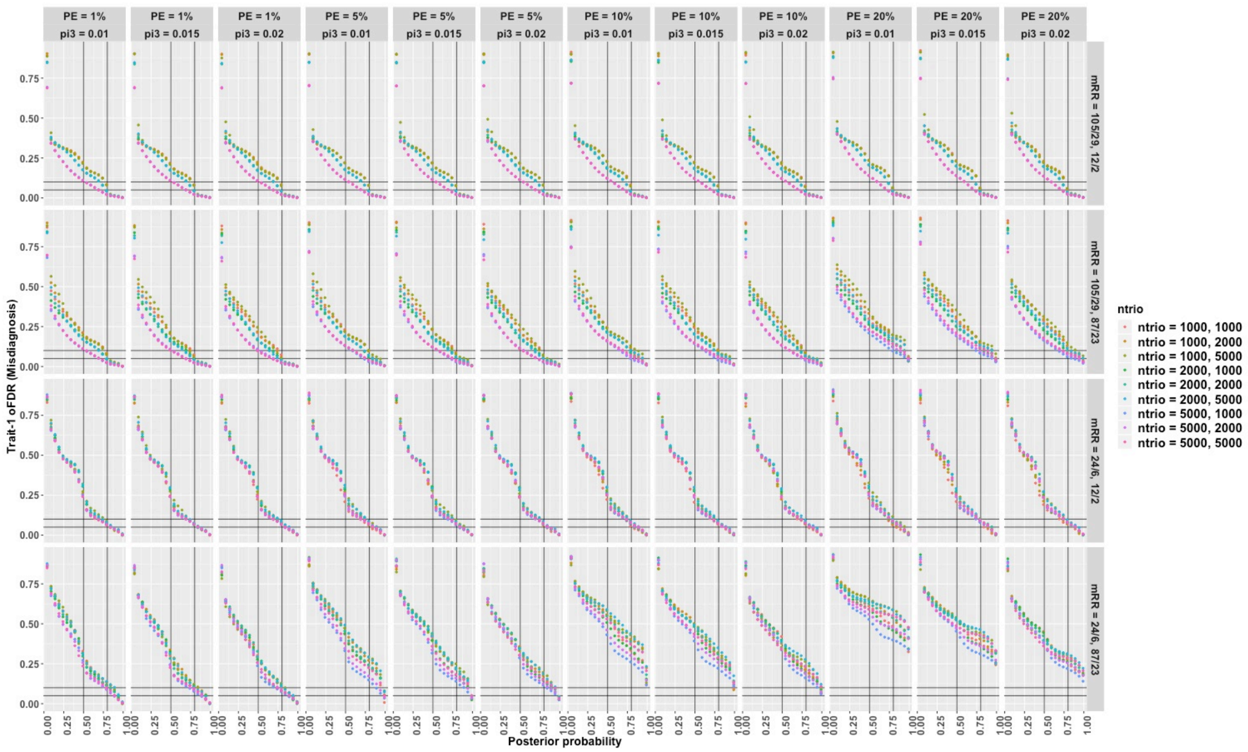

Supplementary Figure 11A

The correlations between observed false discovery rates (oFDRs) and posterior probabilities (PPs) when there are misdiagnosed cases. *These results are for the first-trait group. Genetic/ misdiagnosis information is described in Supplementary Figure 8, 9 and 10. Horizontal lines describe oFDR=0.05 and oFDR=0.1. Vertical lines describe PP=0.5 and PP=0.8.*

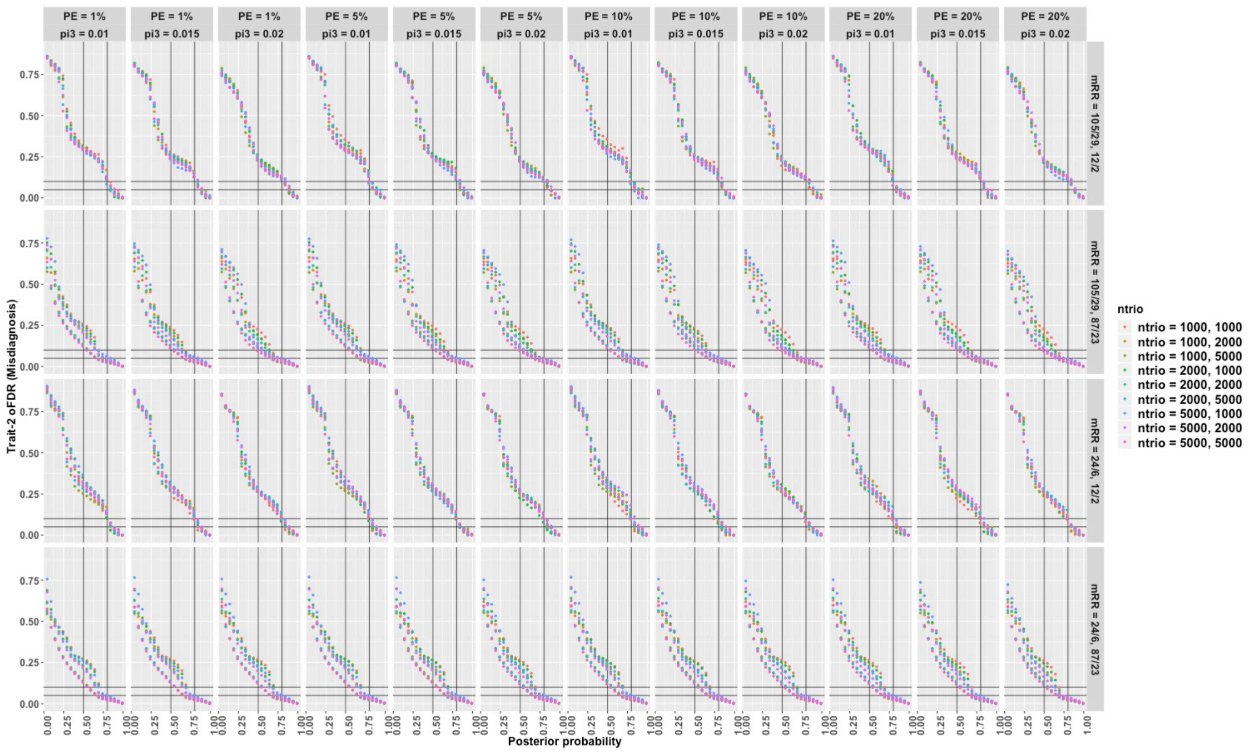

Supplementary Figure 11B.

The correlations between observed false discovery rates (oFDRs) and posterior probabilities (PPs) when there are misdiagnosed cases. *These results are for the second-trait group. Genetic/ misdiagnosis information is described in Supplementary Figure 8, 9 and 10. Horizontal lines describe oFDR=0.05 and oFDR=0.1. Vertical lines describe PP=0.5 and PP=0.8.*

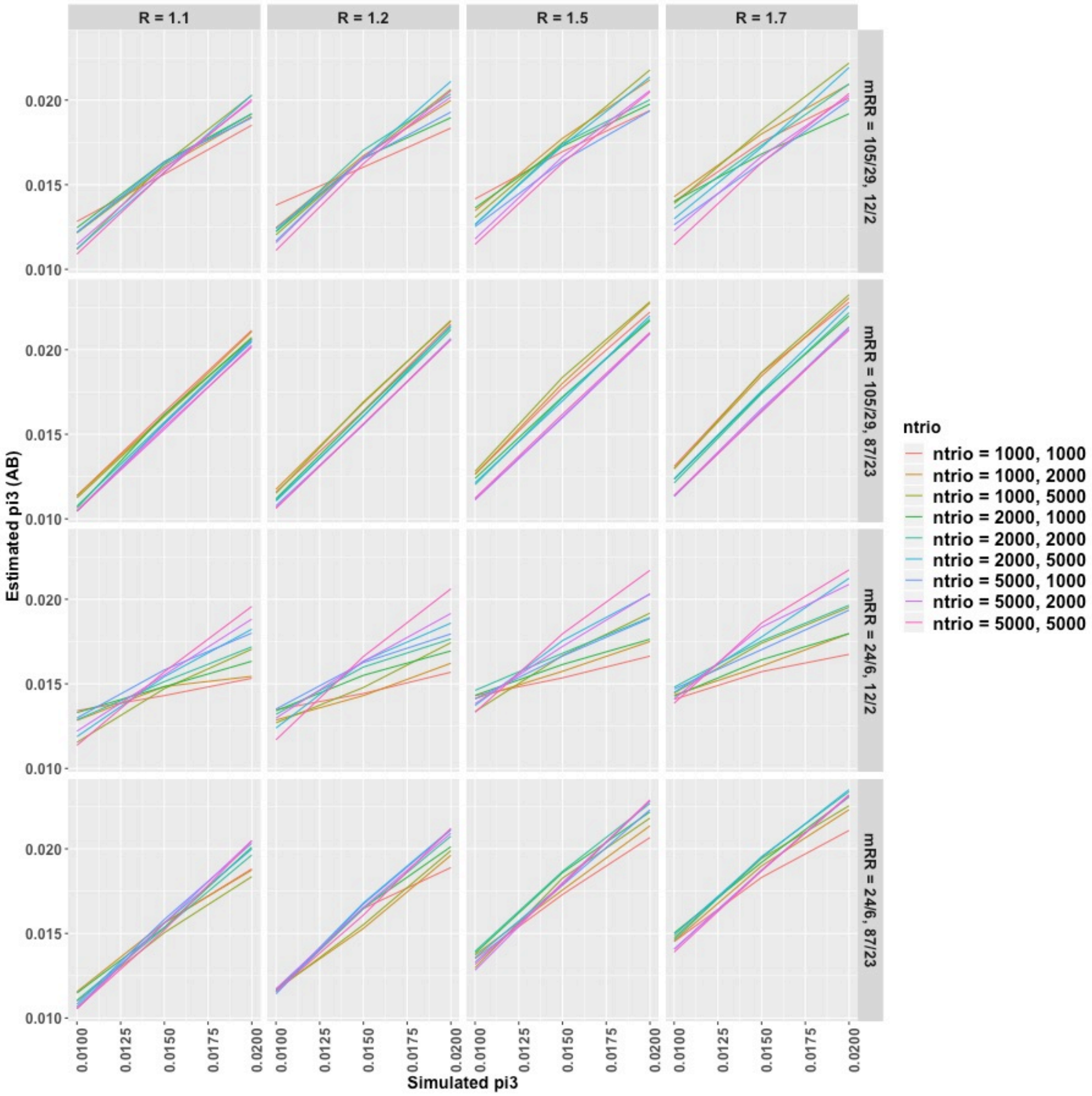

Supplementary Figure 12

The influence of ascertainment bias (AB) on the estimation of $\boldsymbol{\pi_3}$ when there is inflation of gene-level de novo mutations at risk genes of Trait 2. *X-axes show the simulated values of π_3 while Y-axes describe the estimated values of π_3. R: the inflation ratio of DNMs per gene; mRR: mean relative risks; nTrio: the number of parent-offspring trios.*

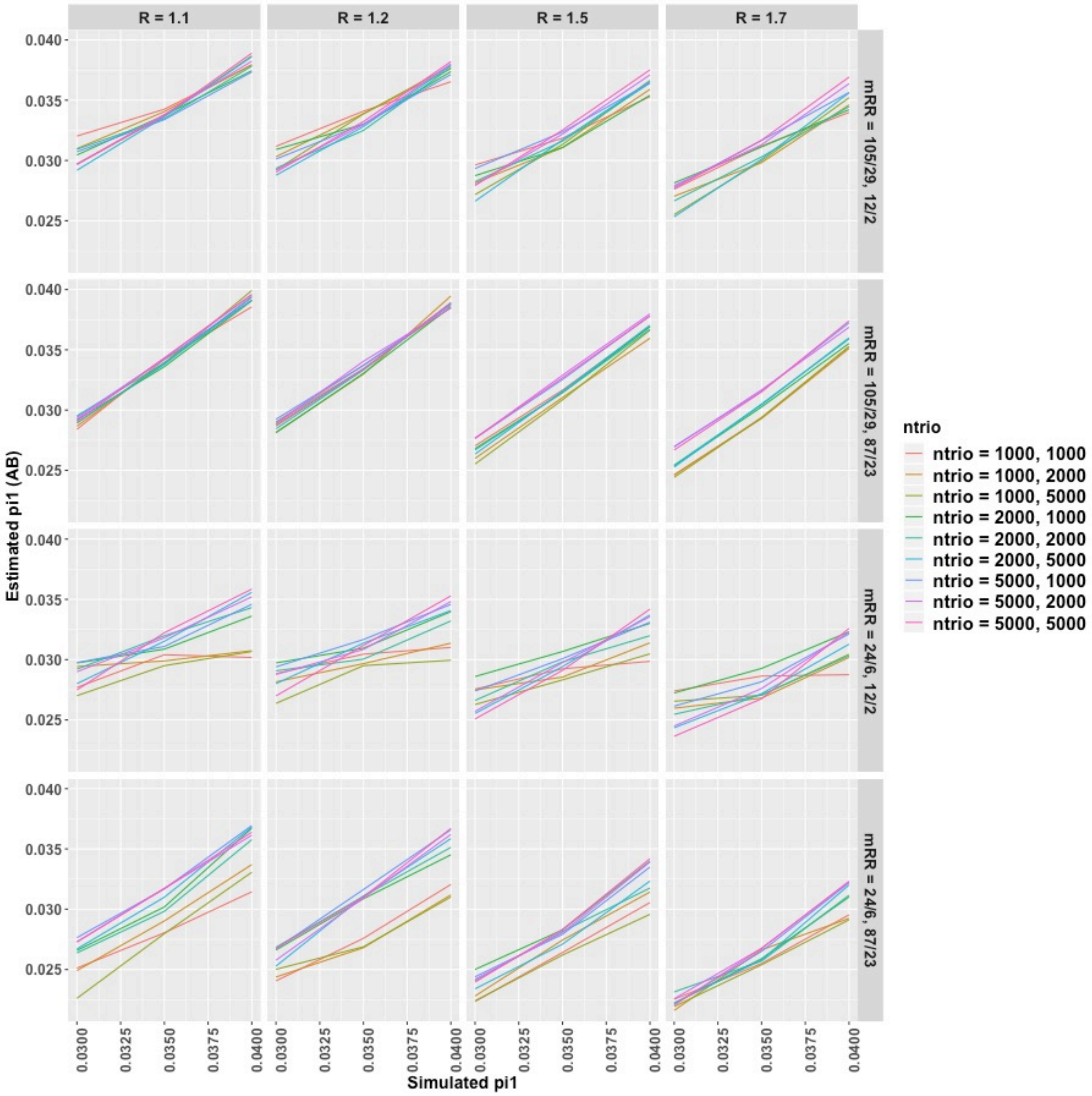

Supplementary Figure 13

The influence of ascertainment bias on the estimation of π_1 when there is inflation of gene-level de novo mutations at risk genes of Trait 2. *X-axes show the simulated values of π_1 while Y-axes describe the estimated values of π_1. R: the inflation ratio of DNMs per gene; mRR: mean relative risks; nTrio: the number of parent-offspring trios.*

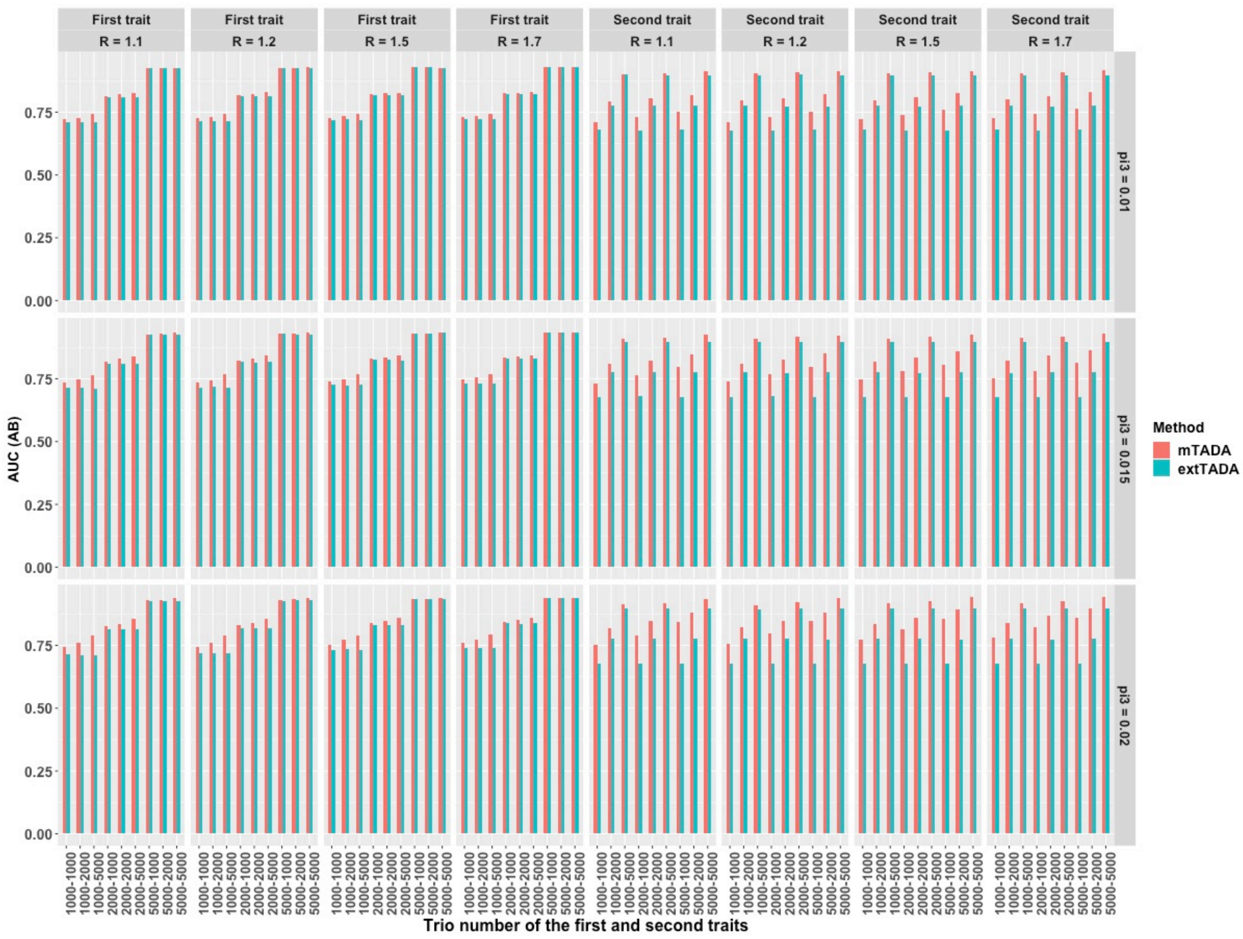

Supplementary Figure 14

Comparison of single-trait and two-trait methods when there is inflation of gene-level de novo mutations at risk genes of Trait 2. *X-axes show the number of parent-offspring trios while Y-axes describe AUC values. R is the inflation ratio and pi03 is the proportion of overlapping genes.*

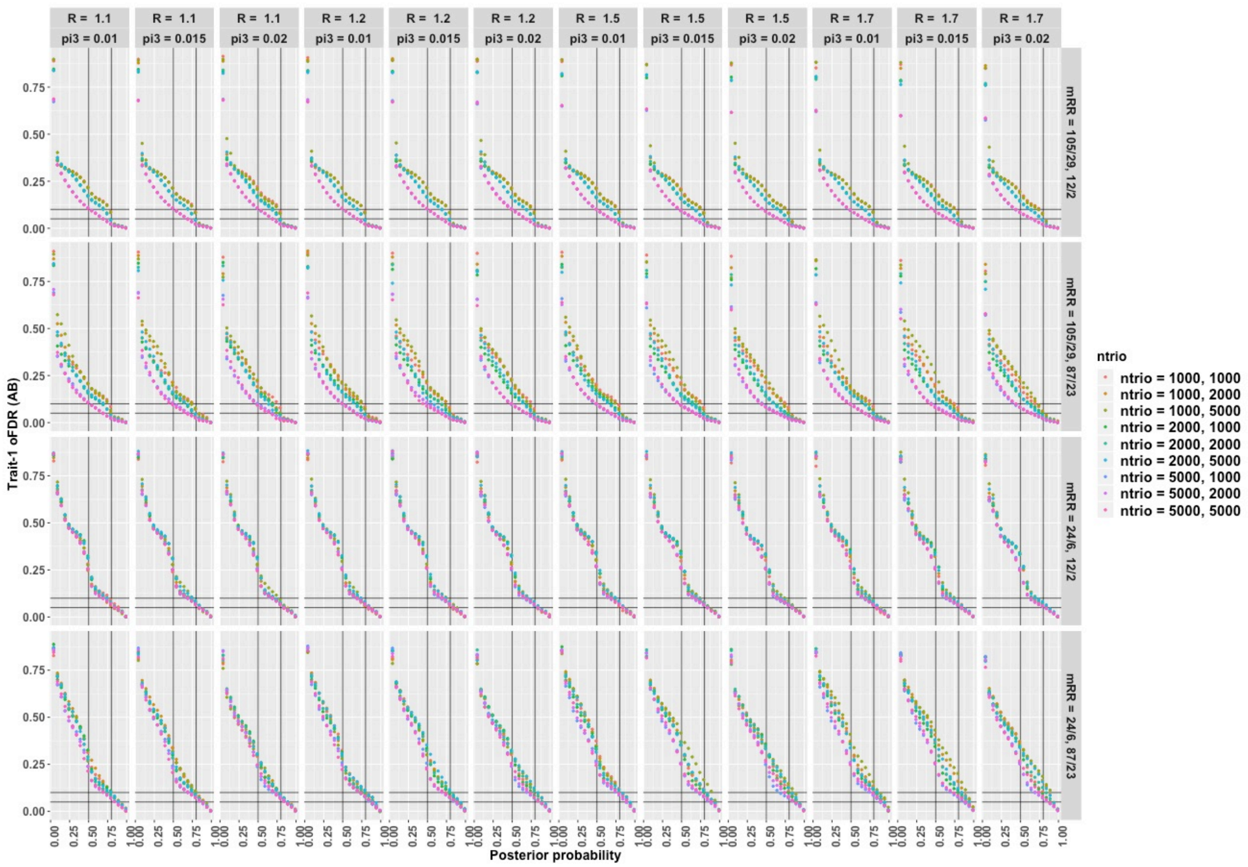

Supplementary Figure 15A

The correlations between observed false discovery rates (oFDRs) and posterior probabilities (PPs) when there is inflation of gene-level de novo mutations at risk genes of Trait 2. *These results are for the first-trait group. Genetic/ascertainment-bias information is described in Supplementary Figure 12-14. Horizontal lines describe oFDR=0.05 and oFDR=0.1. Vertical lines describe PP=0.5 and PP=0.8.*

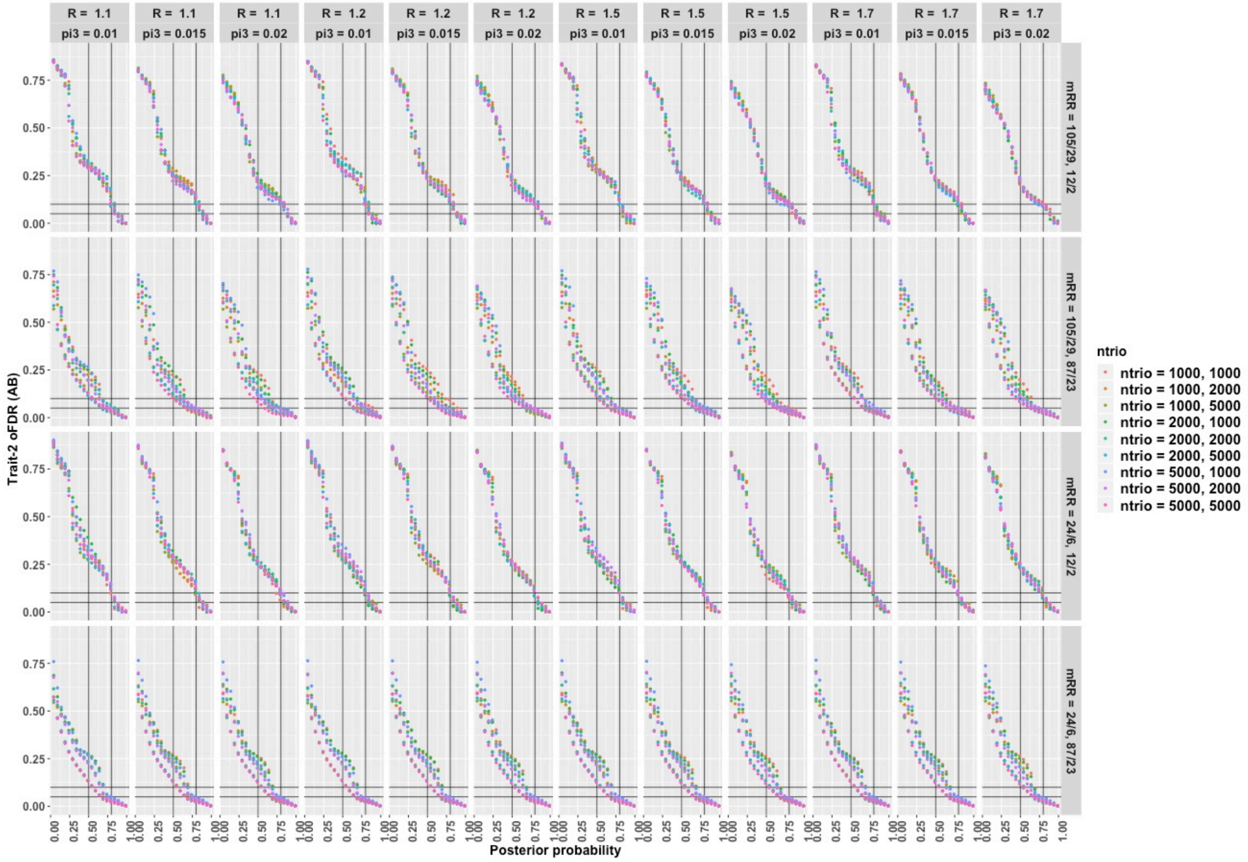

Supplementary Figure 15B

The correlations between observed false discovery rates (oFDRs) and posterior probabilities (PPs) when there is inflation of gene-level de novo mutations at risk genes of Trait 2. *These results are for the second-trait group. Genetic/ascertainment-bias information is described in Supplementary Figure 12-14. Horizontal lines describe oFDR=0.05 and oFDR=0.1. Vertical lines describe PP=0.5 and PP=0.8.*

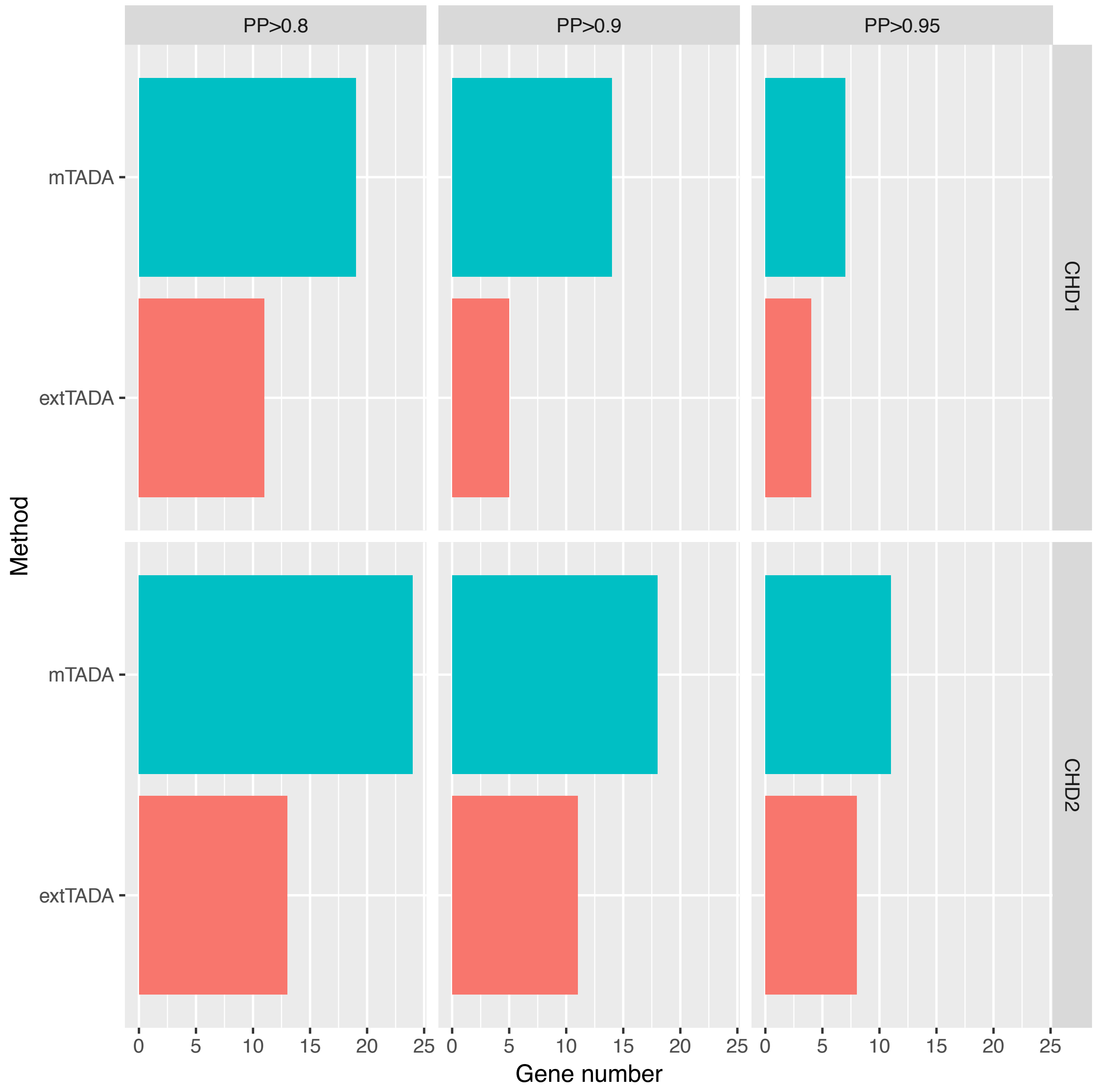

Supplementary Figure 16

Analysis results of two CHD datasets. *The figure describes the number of prioritized CHD genes with different thresholds for mTADA and extTADA. As described in the main manuscript for mTADA, posterior probabilities (PPs) of single traits were calculated as PP1+PP3 and PP2+PP3 for CHD1 and CHD2 respectively.* ***Top pictures****: the information from CHD2 improved the risk-gene identification of mTADA for CHD1.* ***Bottom pictures****: the information from CHD1 improved the risk-gene identification of mTADA for CHD2.*

Reference:

Bahl, E., Koomar, T. and Michaelson, J.J. cerebroViz: an R package for anatomical visualization of spatiotemporal brain data. *Bioinformatics* 2017;33(5):762-763.
Brainstorm Consortium*, et al.* Analysis of shared heritability in common disorders of the brain. *Science* 2018;360(6395).
Cross-Disorder Group of the Psychiatric Genomics Consortium. Genomic Relationships, Novel Loci, and Pleiotropic Mechanisms across Eight Psychiatric Disorders. *Cell* 2019;179(7):1469-1482 e1411.
Cross-Disorder Group of the Psychiatric Genomics Consortium*, et al.* Genetic relationship between five psychiatric disorders estimated from genome-wide SNPs. *Nat Genet* 2013;45(9):984-994.
Gandal, M.J.*, et al.* Shared molecular neuropathology across major psychiatric disorders parallels polygenic overlap. *Science* 2018;359(6376):693-697.
Hammerschlag, A.R.*, et al.* Synaptic and brain-expressed gene sets relate to the shared genetic risk across five psychiatric disorders. *Psychol Med* 2019:1-11.
Nguyen, H.T.*, et al.* Integrated Bayesian analysis of rare exonic variants to identify risk genes for schizophrenia and neurodevelopmental disorders. *Genome Med* 2017;9(1):114.
