# Supplementary material for: mTADA is a framework for identifying risk genes from de novo mutations in multiple traits
Source: Nat Commun. 2020 Jun 10;11:2929. doi: 10.1038/s41467-020-16487-z (PMC7287090; doi:10.1038/s41467-020-16487-z)
Supplement: Supplementary file 4 — Description of Additional Supplementary Files [file 41467_2020_16487_MOESM4_ESM.pdf]

Title: Supplementary Data 1

Description: All results of mTADA for six disorders: schizophrenia (SCZ), congenital heart disease (CHD), epileptic encephalopathies (EE), autism spectrum disorder (ASD), developmental disorder (DD), intellectual disability (ID). Three columns 'BOTH', 'FIRST' and 'SECOND' show the posterior probabilities (PPs) of three models in Figure 1. These three columns describe PP3, PP1 and PP2 respectively in the main text.

Title: Supplementary Data 2

Description: mTADA's results for ID and CHD. These are results of posterior probabilities. The *de novo* mutations are from ID (n trios = 1012) and all CHD datasets (n trios = 2,445).
